# A systematic review of antimicrobial stewardship education for undergraduate students in medicine, nursing, pharmacy, dentistry, veterinary science and midwifery using COM-B framework

**DOI:** 10.1093/jacamr/dlaf245

**Published:** 2026-01-07

**Authors:** Simonne Weeks, Aaron Drovandi, Rebecca Turner, Frances Garraghan, Robert Shorten, Lucie Byrne-Davis, Jo Hart

**Affiliations:** School of Medical Sciences, Faculty of Biology, Medicine and Health, University of Manchester, Manchester, Greater Manchester, UK; British Society for Antimicrobial Chemotherapy, 53 Regent Place, Birmingham, UK; School of Medical Sciences, Faculty of Biology, Medicine and Health, University of Manchester, Manchester, Greater Manchester, UK; School of Medical Sciences, Faculty of Biology, Medicine and Health, University of Manchester, Manchester, Greater Manchester, UK; British Society for Antimicrobial Chemotherapy, 53 Regent Place, Birmingham, UK; Lancashire Teaching Hospitals NHS Foundation Trust, Royal Preston Hospital, Preston, UK; School of Medical Sciences, Faculty of Biology, Medicine and Health, University of Manchester, Manchester, Greater Manchester, UK; School of Medical Sciences, Faculty of Biology, Medicine and Health, University of Manchester, Manchester, Greater Manchester, UK

## Abstract

**Background and Objective:**

Antimicrobial resistance (AMR) is a global health challenge driven by inappropriate prescribing. Antimicrobial stewardship (AMS) education during undergraduate training is important to prepare future healthcare professionals for responsible prescribing, yet provision remains inconsistent across disciplines. To systematically review AMS educational interventions for undergraduate medical, pharmacy, nursing, dental, veterinary and midwifery students, and evaluate the behavioural coverage using the COM-B framework.

**Methods:**

A protocol was registered on PROSPERO (CRD420250655653). Six databases were searched on 4 February 2025. Eligible studies evaluated AMS educational interventions for undergraduate students. Data were independently extracted in duplicate, methodological quality appraised using Medical Education Research Study Quality Instrument (MERSQI) and findings were synthesized narratively using COM-B.

**Results:**

Of 7771 records screened, 42 studies were included, involving 8567 students across six continents. Most were single-group pre-/post-designs, with two randomized controlled trials. All studies addressed psychological capability, mainly by increasing knowledge and reasoning, while reflective motivation was supported in 25/42. Physical opportunity (20/42) and social opportunity (18/42) were less frequent, typically via structured cases or teamwork. Physical capability (9/42) and automatic motivation (2/42) were least represented, usually through simulation, supervised practice or affective engagement. MERSQI scores indicated moderate methodological quality overall.

**Conclusions:**

Undergraduate AMS education is widespread but uneven in its coverage, with emphasis on knowledge and limited attention to skills, opportunities and motivation. Applying COM-B highlights the need for curricula to combine knowledge with rehearsal, authentic resources, teamwork, identity development and positive engagement to prepare graduates for stewardship practice.

## Introduction

Antimicrobial resistance (AMR) has become one of the leading causes of global mortality and imposes an economic burden on healthcare systems.^1^ At the centre of this crisis is inappropriate and suboptimal antimicrobial prescribing, which remains the primary driver of resistance.^2^ In 2019 alone, AMR was directly responsible for 1.27 million deaths and contributed to nearly 5 million additional deaths worldwide.^3^ Since prescribing is one of the most direct and modifiable behaviours influencing resistance, understanding the impact of stewardship education on prescribing is important for preparing future healthcare professionals to address the AMR crisis.

Antimicrobial stewardship (AMS) provides a structured approach to optimizing antimicrobial use and reducing the spread of resistance^4,5^ and the World Health Organization has identified AMS education as a priority for influencing prescribing behaviours and ensuring the responsible use of antimicrobials.^1^ Early exposure to AMS during undergraduate training is therefore important, particularly for students in medicine, pharmacy, nursing, dentistry, veterinary medicine and midwifery, who are involved in both prescribing and wider stewardship roles, covering human and animal health. In this review, undergraduate refers to students still enrolled in their initial degree, including degree-embedded placements and does not include post-graduation, employer-led pre-registration or resident, foundation years. Effective education can build prescribing competence, shape professional identity and instil responsible stewardship practices before graduates assume independent clinical roles.^5^ Without this training embedded in curricula, students risk entering the workforce underprepared to meet stewardship demands.

Despite this recognized importance, AMS education in undergraduate curricula remains inconsistent and fragmented, with many programmes providing limited content or weak alignment with established stewardship competency frameworks.^6–8^ These shortcomings extend beyond medicine and pharmacy to nursing, veterinary and dental education, where students and newly qualified practitioners often report minimal exposure to prescribing guidelines, resources and supervised rehearsal of AMS behaviours.^9–11^ Midwives and dentists are particularly underrepresented in both practice and research, despite longstanding concerns about their preparation in AMS^12^ and much of the existing training continues to emphasize knowledge acquisition rather than developing the capability, opportunity and motivation required to perform AMS-related behaviours in practice.^6,13^

AMS is best understood as a coherent set of actions that promote the responsible use of antimicrobials.^5^ These actions involve appropriate prescribing, de-escalation of therapy, intravenous (IV)-to-oral switch, diagnostic stewardship and patient education and counselling. For undergraduate students, education should therefore be judged not only on what knowledge it imparts but also on how well it prepares them to perform these behaviours when they assume clinical responsibilities. Distinguishing AMS-related behaviours from general learning behaviours is therefore essential for evaluating the effectiveness of educational interventions.

Outcome taxonomies used in health professions education, including Kirkpatrick^14^ summarize reaction, learning, behaviour and results but tend to focus on ‘did it work?’ rather than how or why it worked, assume causal links between levels and often overlook context and unintended effects.^15–17^ Programme evaluation frameworks such as the Context, Input, Process and Product model^18^ address some these issues about examining context, mechanisms and unintended consequences but they operate at a programme level, are time-consuming and do not specify what must changes in learners to produce behaviours.^17,19^ Because stewardship is enacted through behaviour, we anchor this review in COM-B framework, which identifies capability, opportunity and motivation as the necessary conditions for behaviour.^20^ We use COM-B to assess whether interventions target these conditions and report learner outcomes where available, noting that heterogeneity and study designs limit claims about a single most effective method.^15,17^

COM-B defines capability as the knowledge and skills required to act, opportunity as the environmental and social conditions that enable action, and motivation as both conscious intentions and automatic processes that sustain behaviour.^20^ In line with Dyar *et al*.’s definition of stewardship as a set of actions, we use COM-B as an analytic lens to evaluate whether AMS educational interventions develop these behavioural influences during training to prepare students for entry to professional practice.^5^

For clarity, we apply COM-B to the educational setting, treating capability, opportunity and motivation as conditions that curricula can develop during training. We therefore examine students’ fitness for entry to practice in terms of the knowledge, rehearsed skills, simulated or supervised opportunities and motivational influences provided by educational interventions. Undergraduate students cannot yet carry out prescribing behaviours independently, but educational interventions can still cultivate the capability, opportunity and motivation influences so graduates are ready to carry out stewardship within their professional scope. While COM-B has been widely applied to qualified practitioners to identify barriers to stewardship,^21–23^ it has not yet been systematically applied to undergraduate education, making it a novel and appropriate framework for evaluating how curricula prepare students for stewardship practice.

Previous reviews of AMS education have focused narrowly on individual disciplines, national curricula or postgraduate training,^24–28^ yet none have used a behavioural science framework such as COM-B to examine undergraduate AMS education across multiple health professions. This review addresses this gap by systematically applying COM-B to evaluate how current educational interventions support the development of capability, opportunity and motivation for stewardship behaviours among students in medicine, pharmacy, nursing, dentistry, veterinary medicine and midwifery. In doing so, it provides the first cross-professional synthesis of AMS education informed by behavioural science.

This systematic review addresses two research questions. First, what AMS educational interventions are currently delivered to undergraduate medical and healthcare professional students? Second, to what extent do these interventions target the capability, opportunity and motivation that prepare students to optimize antimicrobial use and address antimicrobial resistance?

## Methods

We conducted a systematic review and report in line with PRISMA 2020 guidelines^29^ and registered the protocol with PROSPERO (CRD420250655653).

### Search strategy

After piloting with scoping searches and second-reviewer validation, on 4 February 2025 we searched six databases including PubMed, Web of Science, PsycINFO, EMBASE, CINAHL Plus and Scopus for studies evaluating AMS education among undergraduate healthcare students. The search strategy was based on the PICO framework and informed by previous reviews.^24,30–32^ We applied terms to the title, abstract and keyword fields and included Medical Subject Headings (MeSH) or thesaurus terms where supported. Boolean operators and truncation were used to improve retrieval. The full search strategy is provided in Supplementary file in Table S1 (available as Supplementary data at *JAC-AMR* Online), and an illustrative example is presented in the following:

- {[‘Students, Health Occupations’(MeSH) OR ‘medical student*’ OR ‘nursing student*’ OR ‘pharmacy student*’ OR ‘dent* student*’ OR ‘veterinary student*’ OR ‘midwifery student*’ OR undergraduate OR ‘healthcare student*’] AND
- [‘Antimicrobial Stewardship’(MeSH) OR ‘antibiotic stewardship’ OR ‘antimicrobial resistance’ OR ‘antibiotic resistance’ OR ‘antimicrobial use’ OR antiviral OR antifungal OR antibiotic* OR antimicrobial*] AND
- [‘Knowledge’(MeSH) OR ‘Education’(MeSH) OR interprofessional OR learn* OR evaluat* OR assess* OR teach*]}

We imported all retrieved records into Rayyan^®^ for reference management and duplicate removal.^33^ Titles and abstracts were screened by the lead reviewer (S.W.), with subsets independently reviewed by second reviewers to ensure consistency. Full texts were then assessed against the inclusion criteria. Disagreements were resolved through discussion and consensus with second reviewers (F.G., R.S., R.T.).

### Eligibility criteria

We included peer-reviewed, English-language studies assessing AMS educational interventions delivered to undergraduate students in medicine, pharmacy, nursing, dentistry, veterinary medicine and midwifery. These disciplines were pre-specified in our protocol and informed by previous AMS education reviews.^24,30–32^ Eligible interventions could occur within university settings, clinical placements, rotations or clerkships. These professions commonly contribute to antimicrobial selection and stewardship decisions and, in some countries, also have prescribing rights and inclusion did not depend on prescribing authority. Therefore, we excluded studies involving students from allied health professions such as physiotherapy, optometry, podiatry and physician associate programmes. We also excluded studies on post-graduation interns, residents, junior or foundation doctors, trainees and practicing healthcare professionals to maintain a focus on undergraduate education. In cases where studies included both undergraduate and postgraduate participants, we only included them if the data for undergraduate students were reported separately and could be disaggregated for analysis.

We included studies that evaluated AMS education interventions aimed at improving determinants of stewardship such as, knowledge, attitudes or behavioural intentions through methods such as games, simulations and interprofessional education (IPE)^34^ formats. We considered studies with experimental and quasi-experimental designs, such as pre-/post-intervention studies and cohort studies.

Cross-sectional studies focusing solely on knowledge, attitudes and practices were excluded unless they incorporated an educational intervention.^35^ Studies addressing infection prevention and control without a stewardship focus were excluded in line with AMS-specific inclusion criteria.^36^ We also excluded studies that did not assess at least one of the following learning outcomes such as knowledge retention, antimicrobial prescribing confidence or behavioural intentions. Curriculum mapping and competency development studies were also excluded. No restrictions were applied on year of publication or geographical region, to minimize time-restriction bias and characterize the evolution of AMS education over time.

### Data extraction

We extracted data using a purpose-developed, standardized form that captured study characteristics (year, country, discipline, level of training), intervention design and delivery, outcome measures and main findings. The form was piloted on a subset of studies to ensure clarity and completeness and fields were refined before full extraction. The lead reviewer (S.W.) conducted initial data extraction, which was independently verified by the review team (A.D., F.G., R.S., R.T.). Any discrepancies were resolved by consensus.

### Data synthesis

We conducted a narrative synthesis using the COM-B framework of behavioural influences, conceptualized as the conditions that must be in place for a behaviour to occur.^20^ COM-B subcomponents were operationalized within an educational context.

Capability refers to the knowledge and skills developed through classroom teaching, simulation or supervised practice. In this review, psychological and physical capability were coded as conditions supported by educational interventions rather than as independent performance of prescribing behaviours. Opportunity refers to simulated, supervised or facilitated access to resources and supervised practice including teamwork. Therefore, opportunity was coded as educational conditions that provide rehearsal for, and may enable, future real-world physical and social opportunities. Motivation refers to the reflective and automatic influences supported during education including intentions, professional identity and affective responses. In this case, motivation was coded as conditions shaped during training that may persist or translate directly into prescribing behaviour in workplace conditions. Evidence of capability, opportunity or motivation in authentic clinical environments or educational contexts is reported in the Supplementary coding Table S4 in the Supplementary file and operationalized in Table 1.

**Table 1.** COM-B^20^ subdomains and working definitions applied to AMS undergraduate education

COM-B subdomains	Working definition
Physical capability	The procedural skills required to perform AMS-related tasks in real or simulated clinical environments, such as physical interactions with tools, patients (real or simulated) or biological materials, in accordance with AMS principles.
Psychological capability	The cognitive functions and internal psychological processes required for students to understand, interpret and apply AMS knowledge and reasoning, including attention, comprehension, memory, analytical thinking and behavioural self-regulation, needed for AMS decision making during simulated or supervised practice.
Physical opportunity	The environmental and material factors, e.g. access to AMS guidelines, simulated prescription tools, laboratory results and decision aids, that allow students to practise and demonstrate AMS behaviours in educational settings where they are not yet independently prescribing.
Social opportunity	The interpersonal, interprofessional and cultural enablers that support students’ performance of AMS behaviours through shared norms, peer interaction, mentorship, teamwork and collaborative learning environments within simulations or supervised placements.
Reflective motivation	The conscious beliefs, evaluations, goals and professional identity commitments that shape students’ intention to engage in AMS behaviours.
Automatic motivation	The affective responses, emotional reactions, habits and unconscious tendencies that influence students’ likelihood to perform AMS-related actions.

Each included study was reviewed to identify and code interventions, activities, or outcomes against these operational definitions, with verbatim supporting quotes. Using these definitions the lead reviewer (S.W.) mapped and coded each study to COM-B and L.D., R.T. and J.H. (behavioural science experts) reviewed the mappings and finalized decisions by consensus. Where evidence for a domain was absent, explicit rationale was provided in line with best practice in behavioural intervention reporting^20^ and the full coding table is provided in the Supplementary file, in Table S4. Owing to methodological heterogeneity, spanning pre-/post-designs, cohort studies and randomized controlled trials (RCTs) as well as variation in outcome measurement tools, a meta-analysis was not feasible.^37,38^

### Quality assessment

We assessed methodological quality using the Medical Education Research Study Quality Instrument (MERSQI), which evaluates six domains: study design, sampling, data type, validity of the evaluation instrument, data analysis and study outcomes.^39^ Given that undergraduate students do not prescribe independently, we adapted the study outcomes domain to focus on simulation-based assessments, faculty-observed decision-making exercises or validated AMS knowledge tests rather than direct prescribing behaviours or patient outcomes. Two reviewers independently assessed each study, with discrepancies resolved through discussion to reach a consensus score. The MERSQI tool has been applied in previous systematic reviews of healthcare education interventions.^30,40^ Full details of the adapted scoring system are available in Table S2 in the Supplementary file.

## Results

**Figure 1.**
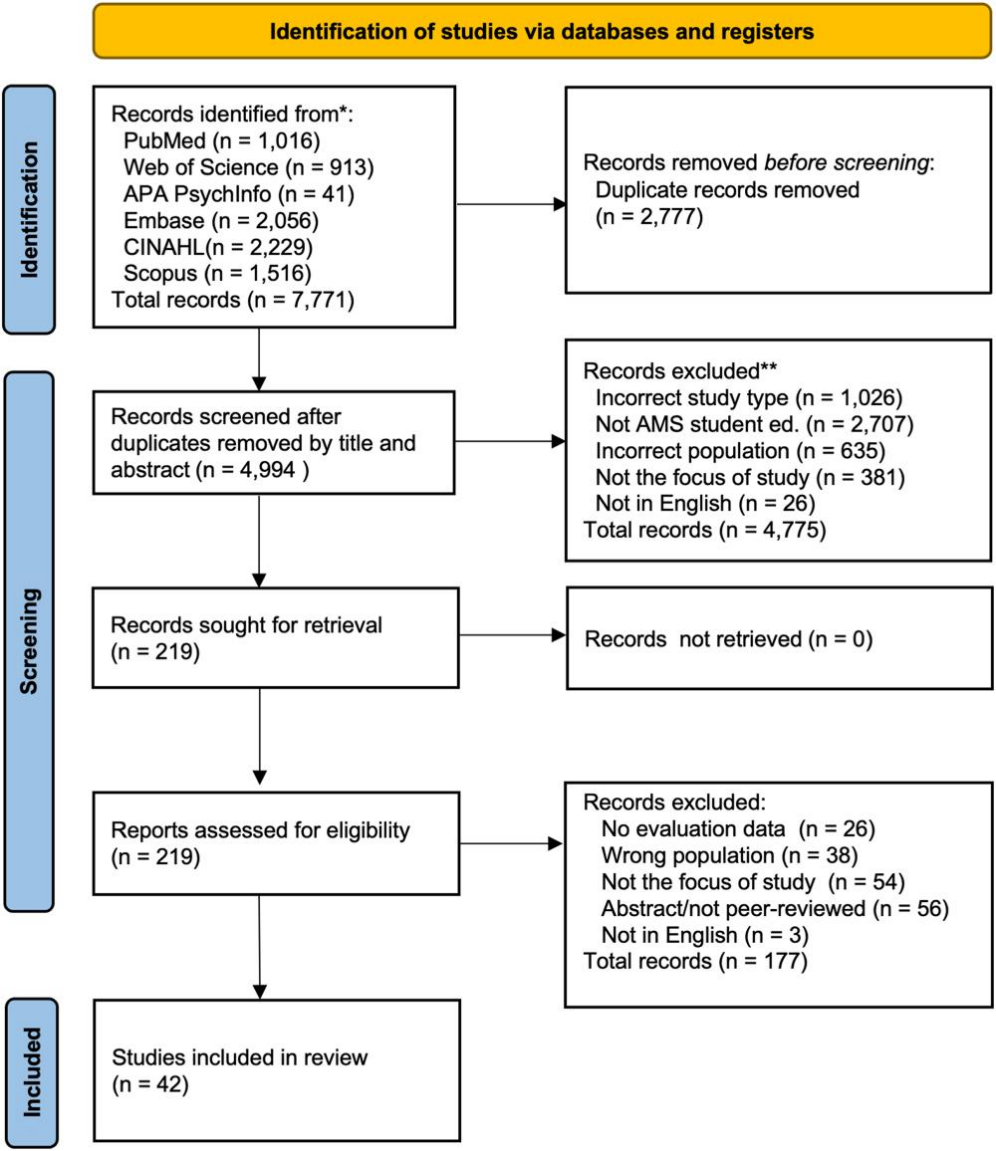
PRISMA^29^ flow diagram for the systematic review on AMS education interventions for medical and healthcare students.

We identified 7771 records and after removing 2777 duplicates using Rayyan software,^33^ and 4994 unique records remained. Following title and abstract screening, we assessed the full text of 219 articles. Screening discrepancies (*n* = 51) arose from studies with an unclear education level or AMS focus and were resolved through reviewer consensus. A total of 42 studies met the inclusion criteria and were included with reasons for exclusion at each stage detailed in the PRISMA flow diagram (Figure 1 and Table S6).

### Study characteristics

The included studies (*n* = 42)^41–82^ were published between 1984 and 2024, with one published in 1984^62^ and the remainder from 2000 onwards. Collectively, these studies involved 8567 students across six continents, predominantly from the USA (19/42)^43,47,48,52,53,55,58,60,62–64,66,68–70,72,77,80,81^ and the UK (5/42).^49,54,67,75,76^ Additional countries represented included India,^78,79^ Egypt,^45,51^ Pakistan^42,56^ and Saudi Arabia^41,65^ (*n* = 2 each), and single studies from Brazil,^59^ Germany,^50^ Iran,^73^ Japan,^44^ Netherlands,^74^ Serbia,^71^ South Korea^57^ and Spain.^82^ One study was conducted across Australia and Malaysia^61^ and another across Austria, England and Italy.^46^

Medical students were most frequently targeted (18/42),^49–51,56,57,59,60,65,69,70,72–75,79–82^ followed by pharmacy (10/42),^44,47,52,53,55,58,61,62,64,68^ dentistry (4/42),^41,45,46,71^ veterinary (3/42)^48,76,77^ and nursing students (2/42).^66,67^ Five studies adopted interprofessional approaches.^42,43,54,63,78^ Detailed characteristics of included studies are presented in Table 2.

**Table 2.** Characteristics of included studies on AMS education for undergraduate students in health and medical disciplines (*n* = 42)

Study (Author, year)	Country	Study design	Population and discipline	Intervention	Comparison group	Key findings
Aboalshamat *et al.*^41^	Saudi Arabia	RCT (parallel groups)	64 students, 4th- to 6th-year, Dentistry	Online course of 9-min video + 25 slides on AMR and prescribing for dentistry	Online course with a 5-min video + 34 slides on oral microorganisms (no AMR content)	Online AMR course increased knowledge but no significant group difference at follow-up
Ahmed *et al.*^42^	Pakistan	Pre–post study	226 students, 4th- and 5th-year Medical and 3rd- and 4th-year Nursing, Medicine/Nursing	6-session, face-to-face, large-group AMR/AMS lectures and pre-/post-knowledge assessments	None reported	Training sessions increased AMR/AMS knowledge for both nursing and medical students
Al Mohajer *et al.*^43^	USA	Pre–post prospective study	125 students, year not reported, Medicine/Pharmacy	Interactive online module, AMS guidelines with 7 core cases on respiratory infections	None reported	Online module improved knowledge but gains declined at 2 months, greatest in pharmacy
Azechi *et al.*^44^	Japan	Pre–post study	234 students, 4th-year, Pharmacy	1-hour pharmacist-led online lecture on AMR, AMS, dosing, pathogens and Japan’s action plan	None reported	On-demand lecture increased pharmacy student knowledge scores pre to post
Badran *et al.*^45^	Egypt	Mixed-methods (Kirkpatrick evaluation)	322 students, 2nd to 4th-year, Dentistry	2-year programme of lectures, group work, role-play and case-based learning on AMS in dentistry	None reported	Knowledge, agreement, satisfaction and AMS behaviours improved; effects sustained at 2-year follow-up
Berr *et al.*^46^	Austria, England, Italy	Pre–post study	39 students, Final-year, Dentistry	Moodle online module, UK antibiotics webcast + virtual patient prescribing game	None reported	Online module increased antibiotic prescribing knowledge by 10% for dental students
Cerenzio *et al.*^47^	USA	Pre–post study	181 students, 3rd-year, Pharmacy	Single-session, face-to-face escape-room workshop on AMS using Google Forms and group discussion	None reported	Interactive game reduced knowledge but high satisfaction and engagement reported
Cole *et al.*^48^	USA	Pre–post study with comparator	238 students, 1st to 4th-year, Veterinary	1-hour online veterinary session: video, case vignettes, SODAPOP mnemonic and scoring rubrics	Received the same as invention group but without the SODAPOP mnemonic	No effect on AMS knowledge and self-efficacy improved on 1 of 4 confidence items
Davies^49^	UK	Pre–post study	36 students, 1st-year, Medicine	10-minute in-class antibiotic ‘Top Trumps^®^’ card game plus slides and interactive recall	None reported	Top Trumps game slightly increased knowledge and 64% rated it useful and enjoyable
Driesnack *et al.*^50^	Germany	Pre–post study	46 students, 4th-year, Medicine	12 90-min hybrid AMS sessions, flipped classroom, gamification, AI, app, podcasts, case discussions	None reported	Gamified AMS course increased knowledge and sustained confidence for empiric choice
El-Sokkary *et al.*^51^	Egypt	Pre–post study	50 students, 3rd-year, Medicine	2-week hybrid etiquette course, interactive sessions, lectures, case scenarios, multimedia reflection	None reported	Knowledge, perceptions, and satisfaction increased and 94% would recommend the elective course
Falcione *et al.*^52^	USA	Pre–post study	45 students, 3rd-year, Pharmacy	5-week elective of lectures, SOAP mnemonic, HPS cases, labs, projects, peer and guided reflection	None reported	Knowledge, confidence, and clinical application improved and 100% agreed AMS understanding increased
Gauthier *et al.*^53^	USA	Pre–post study	22 students, 2nd- and 3rd-year, Pharmacy	15-week hybrid elective of prelecture recordings, flipped classroom, group work, presentations, discussions	None reported	Confidence, preparedness, and satisfaction increased and 95% would recommend course, all objectives met
Guilding *et al.*^54^	UK	Mixed-methods study	157 students, 2nd-year, Medicine/Pharmacy	1-day IPE conference of plenary, workshops, group case learning, event analysis, SimMan sepsis simulation	None reported	Students gained clinical AMS knowledge, valued IPE and reported increased team confidence
Hidayat *et al.*^55^	USA	Pre–post study	48 students, 3rd-year, Pharmacy	4-week pharmacy elective of active learning, mini-lectures, debates, journal club, evidence-based cases	None reported	Awareness, stewardship skills and satisfaction improved and all medians ≥4.0, strong support for active learning
Hussain *et al.*^56^	Pakistan	Pre–post study	500 students, 3rd-year, Medicine	Single-session, face-to-face of lectures, tutorials and small-group AMS discussions	None reported	Self-reported AMS knowledge, attitudes, and interprofessional awareness greatly improved post-session
Jang *et al.*^57^	South Korea	Pre–post study with comparator	109 students, 5th-year, Medicine	1-week face-to-face clinical course of patient record review and parenteral-to-oral conversion tasks	Students who completed the same clinical practice course in infectious diseases at Hanyang University Guri Hospital (they did not participate in the parenteral-to-oral conversion programme)	Parenteral-to-oral programme improved perceived AMS knowledge and switching confidence
Kufel *et al.*^58^	USA	Pre–post-simulation study	143 students, 2nd- and 3rd-year, Pharmacy	3-hour face-to-face session of didactic, student-led interviews, simulated penicillin allergy testing, case studies	None reported	Knowledge, confidence, satisfaction and clinical readiness improved post-simulation with high ratings for format
Laks *et al.*^59^	Brazil	Pre–post study	606 students, 5th- and 6th-year, Medicine	100-hour online course of 5 modules, simulations, live classes, discussion forums and assessments	None reported	Online AMS course increased knowledge, satisfaction and demand for future antimicrobial courses
Larnard *et al.*^60^	USA	Pre–post study	14 students, 4th-year, Medicine	Self-paced video series of 8 whiteboard animations with scaffolded MCQs and antibiotic ladder	None reported	Video series greatly increased knowledge and 100% found videos effective for learning AMS
Lim *et al.*^61^	Australia and Malaysia	OSCE-based evaluation	404 students, 3rd-year, Pharmacy	7-week hybrid of self-directed learning, online lectures, group workshops, 3-station AMS OSCE	None reported	OSCE showed high AMS pass rates and students valued practical application and skill consolidation
MacCosbe and Segelman^62^	USA	Pre–post study with comparator	47 students, 4th-year, Pharmacy	11-week elective on review antibiotic orders, make AMS recommendations, apply review in hospital	Students who did not participate in the elective, only took the required coursework (*n* = 37)	Special problems elective slightly increased antibiotic therapy knowledge for pharmacy students
MacDougall *et al.*^63^	USA	Pre–post study	745 students, 2nd- and 3rd-year, Medicine/Pharmacy	2-hour hybrid of online AMS cases, SDL module, interprofessional workshop with branched-logic cases	None reported	Self-reported AMS knowledge, interprofessional attitudes and perceived value all increased post-curriculum
MacDougall, C.,^64^	USA	Quasi-experimental study with comparator	360 students, 3rd-year, Pharmacy	10-week face-to-face course of active learning, flower diagrams, case exercises, retrieval practice	Traditional lecture-based teaching for other topics within the same course	Active learning increased knowledge and satisfaction and students preferred visual tools for AMS
Malli *et al.*^65^	Saudi	Quasi-experimental study	33 students, year not reported, Medicine	8-hour WHO online course of 15 modules on bacteriology, resistance and appropriate prescribing	None reported	WHO online course increased AMS knowledge, confidence and familiarity from 30% to 100%
Manning *et al.*^66^	USA	Quasi-experimental simulation study	127 students, 2nd-year, Nursing	3-hour virtual simulation of paediatric antibiotic cases, pre-briefing, simulation, debriefing	None reported	Virtual simulation increased AMS knowledge, especially penicillin allergy and stewardship practices
McEwen and Burnett^67^	UK	Pre–post evaluation	167 students, 3rd-year, Nursing	2-hour blended session of lectures, video, group discussions, clinical AMS scenarios for nurses	None reported	AMS understanding rose from 15% to 79% and most students saw AMS as relevant to nursing roles
McGee *et al.*^68^	USA	Pre–post study	33 students, 3rd-year, Pharmacy	12-week face-to-face elective of debates, pre-reading, case vignettes, peer evaluation in AMS	None reported	Debate-based course improved knowledge, confidence and enjoyment; 73% supported wider use
Nori *et al.*^69^	USA	Pre–post study with comparator	183 students, 2nd-year, Medicine	Multimodal hybrid programme of 2 AMS seminars with group work, toolkits and infection prevention	2014 had same as intervention but without the mobile app	Knowledge, confidence, and AMS role perception improved and 99% saw themselves as promoting patient safety
Nori *et al.*^70^	USA	Pre–post study	166 students, 2nd-year, Medicine	2-hour face-to-face seminar of patient/relative stories, expert and CDC/IDSA faculty presentations	None reported	AMS familiarity, understanding and advocacy intent increased, and patient stories were highly impactful
Roganović *et al.*^71^	Serbia	Quasi-experimental study with comparator	64 students, 3rd-year, Dentistry	Mobile app-based SDL of dentalantibiotic.com decision-tree for dental AMS, lectures and practice	Standard teaching without mobile app assistance (no APP group, *n* = 22)	Mobile app group had higher AMS knowledge and 83% reported improved AMS understanding
Rose *et al.*^72^	USA	Iterative intervention study	367 students, Clerkship year, Medicine	12 1-hour in-person session of chart completion, case-based group AMS discussions, iterative feedback	None reported	96% reported improved antibiotic understanding and enjoyment with the active learning session
Sayyadi-Rahaghi *et al.*^73^	Iran	Quasi-experimental study with comparator	132 students, 2nd- and 3rd-year, Medicine	4-month e-learning: weekly video lectures on antibiotic prescribing from Katzung’s pharmacology	2019-year group with face-to-face [control] education delivered by the same professor, using the same content based on Katzung’s textbook. Exam conducted in person	E-learning improved AMS knowledge and satisfaction compared with face-to-face learning
Sikkens *et al.*^74^	Netherlands	Prospective controlled intervention study of long-term effects, combined with randomized controlled intervention study of short-term effects	356 students, 4th-year, Medicine	6-week interactive e-learning: 8 PBL AMS cases, WHO-aligned, exercises, feedback, via MedischOnderwijs.nl	Short-term: 68 interventions, 56 control (standard curriculum). Long-term: 71 interventions, 285 control	E-learning increased OSCE knowledge and prescribing confidence and insecurity halved post-intervention
Stevens *et al.*^75^	UK	Pre–post study	310 students, 3rd-year, Medicine	12-week SDL module: six tutorials + quizzes, 5 case tutorials, face-to-face and online learning	None reported	High quiz scores and satisfaction; only 33% felt module would change future practice
Subasinghe *et al.*^76^	UK	RCT Simulation	119 students, 6th-year, Veterinary	30-minute online simulation: AMRSim 3D veterinary practice, triple-layered video, workshop transcript	Waitlist-control group (*n* = 62) Students did not receive the workshop until after post-test	AMRSim improved knowledge, confidence and motivation for AMS/IPC and 96% intend behaviour
Sun *et al.*^77^	USA	Pre–post prospective study	17 students, 4th-year, Veterinary	Hybrid veterinary AMS curriculum: lectures, lab, clinical case discussions, elective AMS course	None reported	AMS knowledge and confidence remained low post-rotation and slight increase in guideline familiarity
Tamboli *et al.*^78^	India	Pre–post study	178 students, 2nd-year, Medicine/Dentistry/Nursing	2-hour face-to-face session: lectures on AMR and prescribing, pre–post- knowledge test, all disciplines	None reported	Educational session improved knowledge and attitudes for medical, dental and nursing students
Tirupakuzhi *et al.*^79^	India	Pre–post study	542 students, 4th-year, Medicine	4-week online course: 20 short expert-led lectures on AMS, microbiology and infection management	None reported	Online lectures increased AMS knowledge across microbiology, pharmacokinetics and infection management
Tolloch *et al.*^80^	USA	Mixed-methods evaluation	168 students, 2nd-year, Medicine	2 120-min TBL modules: readings, webcasts, readiness tests, group clinical scenario discussions	None reported	Mixed TBL perceptions with team activities rated positively, webcasts/content difficulty less favoured
Wang *et al.*^81^	USA	Pre–post study	18 students, 3rd- and 4th-year, Medicine	2-week hybrid elective: small-group didactics, e-learning, audit-feedback, AMS rounds, interprofessional	None reported	Comfort with prescribing, AMS and teamwork rose from <40% pre- to 100% post-course
Yuste *et al.*^82^	Spain	Pre–post study with comparator	994 students, 1st to 6th-year, Medicine	In-person infectious diseases course: lectures on AMR, stewardship, cultures, therapy, group comparisons	Group 1 (students without training in infectious diseases)	AMS training improved knowledge and perceptions, although knowledge declined 1-year post-training

IPC, infection prevention and control.

**Figure 2.**
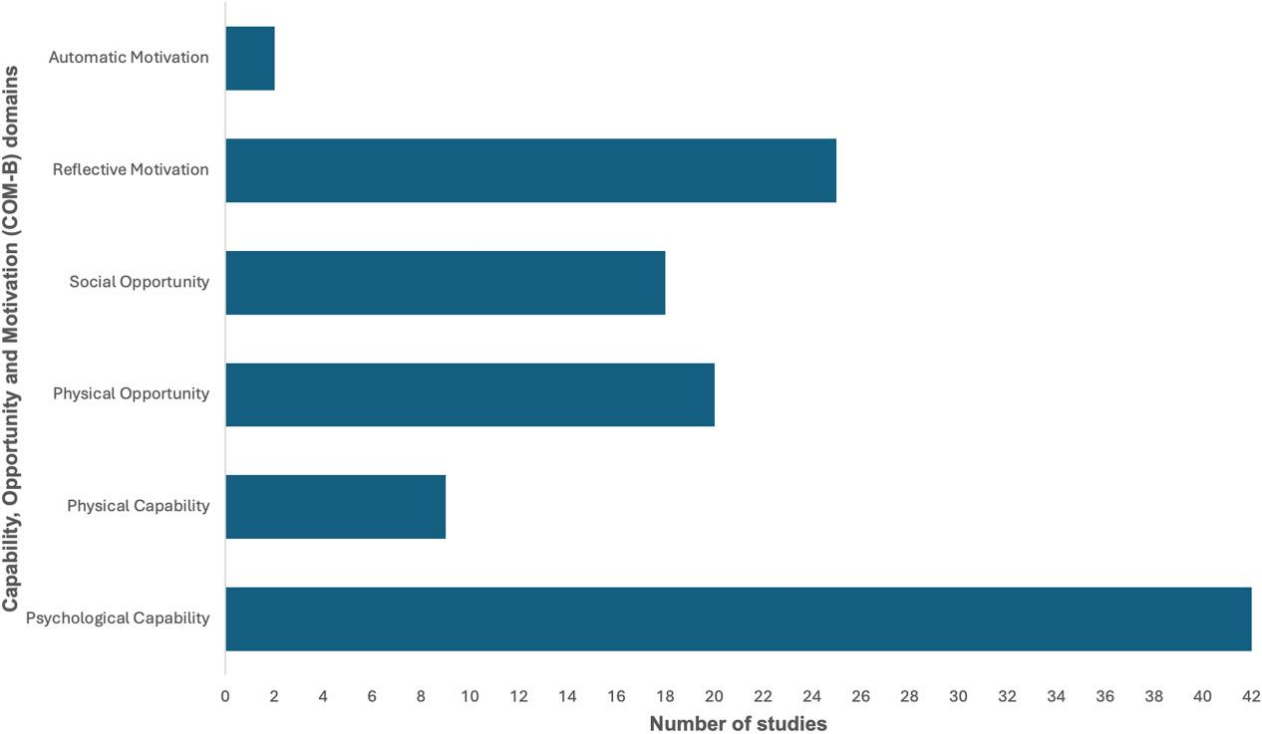
The number of studies providing the conditions for capability, opportunity and motivation (COM-B) in AMS undergraduate education, which represent the behavioural influences necessary for future AMS practice.

We present findings by COM-B domain and indicate the student audience for each example so readers can see where intervention design reflects expected responsibilities at entry to practice. In assessing how educational interventions targeted the COM-B conditions, we coded all studies providing psychological capability influences (42/42)^41–82^ with over half targeting reflective motivation (25/42).^45,48,50–54,56,58,60–63,65,67–71,74–76,80–82^ Physical capability (9/42)^45,52,54,57,58,61,62,74,81^ and automatic motivation (2/42)^47,71^ were the least frequently supported influences, while physical (20/42)^45,47,50,52,54,55,57,58,61,62,64,66,68,69,71,74–76,81^ and social opportunity (18/42)^45,47,52–55,57,58,61–63,66,68,69,72,76,80,81^ were less often targeted, as illustrated in Figure 2.

### Psychological capability (42/42)

All included studies addressed psychological capability, defined as the knowledge, reasoning, and decision-making conditions that support students in knowing how to perform stewardship behaviours. Two approaches were evident, with some interventions reinforcing factual knowledge (20/42)^41–46,49,51,56,58–60,64,66,70,73,76,78,79,82^ and others extending into case-based reasoning or decision making (22/42).^47,48,50,52–55,57,61–63,65,67–69,71,72,74,75,77,80,81^ Where both elements were present, studies were categorized under reasoning, reflecting our definition that prioritizes the application of knowledge as a behavioural influence, rather than evidence of behaviours themselves.

Among the 20 knowledge-focused interventions, lectures and online modules were most common, usually assessed with pre-/post-tests. These interventions strengthened recall of AMS knowledge but did not provide the conditions for students to carry out stewardship behaviours. Short courses frequently produced immediate but short-lived gains. For example, both an online course for Saudi dental students and a US module on respiratory infections raised knowledge scores initially, although these were not sustained at 2 months.^41,43^ By contrast, more sustained improvements were reported in extended formats, including multi-session lectures in Pakistan and e-learning courses in Brazil and Iran.^42,59,73^ Innovative formats such as prescribing games, card games and schematic ‘flower diagram’ teaching supported short-term recall knowledge that would guide prescribing behaviour but did not provide opportunities to perform those behaviours.^46,49,64^

Of the 22 reasoning-focused studies, two illustrate how interventions extended psychological capability into decision-making skills that underpin stewardship behaviours. For example, US veterinary students applied AMS principles across nine scenarios yet continued to struggle with interpreting sensitivity data.^77^ By contrast, a German gamified course embedded case-based vignettes with feedback, reinforcing reasoning about how to act in realist AMS contexts.^50^

It is worth noting that 12 studies (29%)^41–44,46,49,59,64,73,77–79^ addressed only psychological capability, while the remainder combined it with other COM-B domains (opportunity and motivation). This shows that factual understanding was supported across all studies but sometimes as the sole influence.

### Physical capability (9/42)

Nine studies targeted physical capability, defined as the hands-on conditions and skills developed during education that prepare students to perform stewardship behaviours in supervised or simulated contexts.^45,52,54,57,58,61,62,74,81^ Two approaches included structured simulation or OSCE exercises (5/9)^52,54,58,61,74^ and supervised clinical practice (4/9).^45,57,62,81^

Simulation formats varied from hybrid laboratory and bedside tasks for example, microbiology assay interpretation with human patient simulation^52^ to high-fidelity sepsis and OSCE scenarios.^54,61,74^ Others placed stronger emphasis on building the skills needed for patient-facing tasks, such as conducting allergy interviews or penicillin scratch testing.^58^ Together, these examples illustrate that simulations created educational conditions in which students could rehearse competencies required for AMS behaviours such as prescribing, diagnostic and patient communication, but not carry them out independently.

By contrast, clinical practice-based interventions embedded stewardship behaviours into healthcare workflows. Here, educational interventions created capability conditions by allowing students to engage with real patient data, review antibiotic orders, recommend therapy modifications or write prescriptions during routine care, with supervisors intervening to confirm or correct decisions.^45,57,62,81^ These activities represented rehearsal of behaviours under supervision.

Although these nine interventions helped to develop students’ capability for performing practical AMS tasks, they were relatively uncommon compared with the emphasis on knowledge and reasoning. All these studies also create physical opportunity conditions by providing students with access to records, prescribing resources or supervisory support, highlighting a connection between capability (knowledge and skills) and opportunity (availability and access of resources and teamwork).

### Physical opportunity (20/42)

Twenty studies created physical opportunity conditions, defined as the provision of resources, tools or structured environments that enabled students to rehearse stewardship behaviours under supervision.^45,47,48,50,52,54,55,57,58,61–63,66,68,69,71,74–76,81^ Structured clinical cases and resources were the most common format (12/20)^45,47,52,54,55,58,61,63,68,74–76^ while digital or gamified tools were less frequent (5/20).^48,50,66,69,71^ A further three interventions used patient data to create opportunity, however, since these required students to attempt supervised performance of stewardship behaviours, they are summarized under the physical capability section.^57,62,81^

Structured clinical cases and materials created the conditions where students had the opportunity to have access to patient data, laboratory culture and susceptibility results, or prescribing charts, which students could use to rehearse AMS-related decisions.^45,47,52,54,55,58,61,63,68,74–76^ Illustrative examples include OSCEs where students practice prescribing through simulation,^74^ online cases that scaffolded the initial antimicrobial choice to targeted therapy using embedded laboratory findings,^75^ and classroom scenarios in which students justified treatment plans using reference materials.^55,68^

A smaller group used digital or gamified tools which offered opportunities for students to be exposed to external resources needed for stewardship behaviours in future practice.^48,50,66,69,71^ For example, Serbian dental students applying a decision-tree mobile app in practice sessions^71^ and German medical students received AI-generated feedback on anonymized microbiology records in a gamified course.^50^ Other interventions created a space for students to engage with stewardship resources such as a metacognitive framework applied to select antimicrobials from simulated susceptibility cases^48^ and US nursing students working through virtual patient simulations.^66^

### Social opportunity (18/42)

Eighteen studies created social opportunity, defined as collaborative or supervised context that supported students to develop the ability to perform stewardship behaviours collectively.^45,47,52–55,57,58,61–63,66,68,69,72,76,80,81^ Three approaches were identified that included, peer or small-group work (7/18),^45,47,53,55,68,69,72^ interprofessional teamwork (3/18)^54,63,81^ and mentorship or expert modelling (4/18)^57,62,76,80^ with a further four combining elements of all three.^52,58,61,66^

The most common strategy was peer or small-group work where interventions supported students to co-produce stewardship plans or solve cases together.^45,47,53,55,68,69,72^ For example, US pharmacy electives used debates that mirrored ward rounds, requiring teams to rehearse decision-making processes involved in prescribing and justify antimicrobial choices.^55,68^ Escape-room activities also allowed collaboration by having students interpret microbiology data in teams, though learning gains were limited.^47^

Interprofessional teamwork was less common but provided opportunities for students to appreciate how stewardship is distributed across roles.^54,63,81^ For example, UK medical and pharmacy students collaborated during a sepsis workshop on prescribing simulations.^54^ Similarly, US medical and pharmacy students worked together on branched-logic AMS cases.^63^ These activities exposed students to the collaborative processes that underpin stewardship in practice.

A key aspect of four studies was mentorship and expert modelling through supervision and immediate feedback.^57,62,76,80^ A couple of examples include veterinary students in the UK describing how facilitator feedback during a simulation prompted reflection and commitment to safer practice,^76^ while US medical students in team-based learning exercises defended antimicrobial choices under faculty guidance.^80^ These interventions created supervised conditions that allowed students to see how stewardship behaviours are performed in real settings.

Several electives and OSCEs combined these approaches, blending peer collaboration, interprofessional teamwork and expert supervision. Examples included pharmacy electives using patient simulation,^52^ allergy assessments,^58^ OSCE stations^61^ and nursing simulations.^66^

When taken as a whole, social opportunity was most often created through peer collaboration, with fewer interventions embedding interprofessional teamwork or expert mentorship. Outcomes generally indicated stronger teamwork attitudes and confidence in AMS-related tasks.

### Reflective motivation (25/42)

A total of 25 studies addressed reflective motivation, defined as the conscious beliefs, intentions, goals and professional identity that act as motivational influences shaping students’ deliberate commitment to stewardship behaviours.^45,48,50–54,56,58,60–63,65,67–71,74–76,80–82^ Evidence was organized into four categories: confidence and self-efficacy (11/25),^45,48,50–52,58,60,61,65,74,81^ professional role and identity (7/25),^53,54,63,67–69,80^ intentions or action plans (4/25)^62,70,71,75^ and outcome expectancies (3/25).^56,76,82^ Several studies contributed to more than one influence.

A recurring pattern was improved confidence to prescribe and manage antibiotics responsibly. Eleven studies^45,48,50–52,58,60,61,65,74,81^ reported increased self-efficacy creating conditions that supported students’ belief that they could perform stewardship tasks in future practice. For example, Egyptian medical students reported preparedness to interpret antibiograms^51^ and US students described greater comfort in selecting antibiotics after video-based training.^60^

Another influence was professional role and identity, where interventions shaped students’ recognition of stewardship as part of their future professional responsibilities. This was evident in a study involving UK nursing students who linked AMS to their responsibility to question prescriptions during placements,^67^ while US medical students affirmed their stewardship role after app-based teaching.^69^

Four studies went further, with students outlining intentions to apply stewardship in future practice.^62,70,71,75^ For example, in Serbia, dentistry students reported plans to continue using mobile prescribing apps,^71^ whereas Irish medical students indicated that case-based training would alter their future prescribing.^75^ These findings suggest that in some contexts, knowledge gains translated directly into deliberate action plans but again, these represent motivational influences developed during education, not evidence of behaviours being carried out.

Finally, three studies connected stewardship behaviours to anticipated benefits for patients and health systems.^56,76,82^ Pakistani medical students associated AMS with reducing resistance and lowering healthcare costs.^56^ Spanish medical students linked intravenous-to-oral switching with improved care.^82^ UK veterinary students described avoiding unnecessary antibiotics after recognizing risks of previous behaviours.^76^

Overall, the evidence here indicates that reflective motivation was supported through gains in self-efficacy and professional role identity. Fewer interventions encouraged students to form explicit action plans or outcome expectancies, but where present, these reinforced commitment to stewardship in practice. It is worth remembering that these educational interventions shaped motivational influences related to students’ readiness and intention to adopt stewardship behaviours, but not the behaviours themselves.

### Automatic motivation (2/42)

Only two studies addressed automatic motivation, defined as the emotions or unconscious influences that shape students’ likelihood of engaging in AMS behaviours.^47,71^ These motivational influences were expressed as either negative arousal or positive satisfaction during AMS-related tasks.

In one intervention, nearly half of students reported stress or feeling overwhelmed during a time-pressured escape room.^47^ This suggests that competitive or high-pressure environments, while engaging created conditions that inadvertently heighten anxiety. By contrast, Serbian dental students described a decision-tree prescribing app as ‘simple’ and ‘satisfying’, suggesting that positive affective responses functioned as motivational influences that reinforced engagement.^71^

These examples highlight that affective responses were rarely captured during the evaluations of educational interventions. Where present they showed that the emotions students experienced during AMS activities were motivational influences that could shape whether stewardship behaviours are carried out. For example, feeling stressed or enjoying a task is different from prescribing an antibiotic, but such feelings represent the conditions that can influence whether prescribing occurs, and how effectively it is performed.

### Quality of included studies

MERSQI scores were calculated for each included study to assess methodological quality and risk of bias.^39^ Consensus between two independent reviewers was achieved for all scoring decisions. Agreed total scores ranged from 7.0 to 15.0 (mean = 10.0, SD = 1.65, maximum possible score = 18), with most studies demonstrating moderate methodological quality. The strongest performance was observed in the study design and data analysis domains, reflecting the frequent use of pre–post or quasi-experimental designs and appropriate statistical analysis methods. By contrast, greater variability was noted in the validity and sampling domains, where studies often lacked detailed reporting on instrument validation or relied on single-site samples with low response rates. Higher domain scores were achieved when studies included simulation-based tasks, faculty-observed decision-making exercises, or validated AMS knowledge assessments. Full details are available in the Supplementary file, in Tables S3 and S5.

## Discussion

This systematic review identified 42 studies evaluating AMS educational interventions delivered to undergraduate students in medicine, pharmacy, nursing, dentistry, veterinary medicine and midwifery across six continents. Collectively involving >8500 students, the majority of interventions focused on strengthening psychological capability through knowledge and reasoning, with more than half also targeting reflective motivation. Influences related to physical opportunity (resources, environments) were addressed in half of the studies, while social opportunity (teamwork, supervision) were in fewer than half. Physical capability was supported in fewer than one in four interventions, usually through simulations or supervised practice, and automatic motivation was rarely considered. These findings highlight that AMS education is widespread and methodologically diverse, yet coverage across COM-B influences remains uneven. Descriptively, most concrete examples of skills rehearsal and supervised practice with authentic resources arose in medicine and pharmacy cohorts, whereas the smaller dentistry, veterinary and nursing samples more often reported knowledge and confidence outcomes. However, these patterns across the student groups should be interpreted cautiously given the unequal number of studies across the disciplines.

By applying the COM-B behavioural science framework, this review shows that AMS education interventions are most likely to prepare students for effective stewardship practice when they simultaneously provide cognitive knowledge and reasoning (psychological capability), create conditions for rehearsing procedural skills (physical capability), offer authentic resources (physical opportunity), embed teamwork and supervision (social opportunity), strengthen professional identity and intentions (reflective motivation), and support positive emotional engagement (automatic motivation). Implementation of these conditions may differ by profession, so we report by COM-B domains across the student cohort rather than ranking disciplines.

### Limitations of the review

Before further interpreting these findings, it is important to acknowledge several limitations of this review. First, there was considerable heterogeneity among the included studies, with differences in intervention types, populations, outcome measures, study designs and publication dates. Owing to this variability a meta-analysis was not deemed appropriate and therefore this limited direct comparisons of intervention effectiveness. We addressed this by conducting a narrative synthesis using the COM-B framework^20^ to systematically map the key behavioural influences supported by AMS educational interventions. Using this framework was a slight deviation from our original protocol but it provided a reliable and validated structure to examine the variation in how interventions create conditions for capability, opportunity and motivation.

Second, the methodological quality of the included studies was variable. Many relied on pre-/post-designs without control groups and often measured influences such as knowledge or confidence using self-reported and non-validated instruments. We looked to minimize bias by systematically assessing study quality with the MERSQI instrument,^39^ however, limitations in the primary evidence base remain and may affect the reliability of some findings.

Third, our review was restricted to published, peer-reviewed articles in English. This introduces a risk of publication and language bias, which may have led to the exclusion of relevant studies published in other languages or grey literature. While a comprehensive search strategy was used across multiple databases, it is possible that some studies were missed.

Another limitation is that we did not account for differences across professions and countries in legal prescribing responsibilities, and emphasis placed on prescribing within curricula. These differences limit direct cross-country and cross-professional comparisons of prescribing competence. As a result, we summarize findings across disciplines but do not compare or rank professions or claim a single most effective educational approach. Our COM-B focus partly mitigates this by comparing educational conditions that can shape behaviours rather than observed prescribing behaviours.

A further conceptual limitation is that COM-B was applied here to educational settings rather than clinical practice. Capability, opportunity and motivation were therefore coded as influences created during training, such as supervised practice, simulated cases or access to stewardship resources, rather than as actual prescribing behaviours. While this adaptation does not capture behaviour in practice, it is valuable because it shows how curricula can provide the conditions that build capacity and competence for stewardship before students take on clinical responsibility.

### Strengths of this review

While these limitations should be considered, this review has several important strengths. To our knowledge, this is the first systematic review to adapt and apply the COM-B framework to synthesize AMS educational interventions across all major undergraduate medical and healthcare professions.^20^ This approach allowed us to systematically identify and map the behavioural influences of capability, opportunity and motivation, supported by educational interventions, providing a comprehensive understanding of how AMS education prepares future prescribers. Second, we conducted a systematic and rigorous search across six major databases, capturing a wide range of studies from six continents and multiple health and medical disciplines. This broad scope increases the generalisability of our findings and ensures that the synthesis reflects diverse educational contexts. Finally, by explicitly focusing on behavioural influences, this review offers actionable insights for curriculum development and guides future research to better align AMS education with the competencies required for effective stewardship practice.

### Findings in context of existing literature

Consistent with earlier reports,^1,13^ our review confirms that undergraduate curricula across medicine, pharmacy, nursing, dentistry and veterinary training remain inconsistent. These findings are in line with previous studies documenting persistent gaps in AMS teaching, particularly in the UK and USA, where alignment with stewardship competencies remains weak,^6–8^ and are further supported by reports of similar deficiencies in veterinary and dental education.^9–11^ Our review also supports earlier observations that midwives and dentists remain underrepresented in AMS education research, echoing concerns identified more than a decade ago.^12^

In agreement with previous reviews, we found that most interventions primarily supported psychological capabilities, especially knowledge and reasoning processes that underpin AMS-related decisions.^24,27,28,31^ However, our findings extend the existing literature by mapping the limited support for practical skills development, opportunities and motivational influences within these programmes, which are areas that have not been systematically synthesized across professions until now. This extension is particularly important given that gaps in practical skills, social supports and motivational influences leave newly qualified healthcare and medical graduates underprepared for the workplace challenges, such as diagnostic uncertainty, time constraints, hierarchical pressure and patient expectations.^25,83^ These contextual influences on prescribing are not always addressed in undergraduate education, as previous studies have identified.^25,83^ Approaches to AMS differ across countries and these differences probably influence COM-B conditions, especially opportunity and motivation.^6–11,25,83^

### Implications of the results

The findings of this review highlight the need for future research that goes beyond knowledge-based AMS education to address the influences of practical skills, social influences and motivational conditions that must be in place for AMS behaviours in clinical practice. Table 3 summarizes the behavioural gaps identified across the COM-B influences and provides recommendations for future curriculum development and research.

**Table 3.** Behavioural influence gaps in AMS education and recommendations for curriculum development

COM-B domain	Identified gap	Recommendations with examples
Psychological capability (knowledge and reasoning)	Most interventions reinforced factual knowledge but fewer extended to reasoning and decision making.	Combine short lectures with structured clinical reasoning exercises e.g. case vignettes requiring empiric–directed therapy decisions and scaffolded OSCE stations testing antibiotic switch decisions.
Physical capability (practical skills)	Few opportunities were created for students to rehearse prescribing or diagnostic skills in supervised settings.	Integrate AMS-focused OSCEs and simulations e.g. prescribing from drug charts, interpreting antibiograms, allergy testing, switching IV–oral therapy, with feedback from faculty or clinical preceptors.
Physical opportunity (resources and environments)	Structured cases were common, but fewer interventions provided access to authentic resources or digital decision-support tools.	Embed real-world AMS tools into curricula e.g. use of hospital antibiograms, prescribing charts, decision-tree mobile apps, stewardship alerts so students practice in contexts mirroring clinical settings.
Social opportunity (teamwork and supervision)	Limited interprofessional teamwork with supervision and expert modelling underused.	Design interprofessional teaching (medicine, pharmacy, nursing, dentistry and veterinary where relevant) using ward-round style cases and team-based simulations and include direct supervision/feedback from infectious disease or AMS specialists.
Reflective motivation (identity, intentions, goals)	Many interventions supported confidence but fewer strengthened role identity or future action plans.	Incorporate structured reflection and action-planning e.g. portfolio entries on AMS responsibilities, sessions where students define their stewardship role in practice and commitment-to-change contracts or pledges.
Automatic motivation (emotions and affective responses)	Rarely evaluated with some activities caused stress, while others generated positive engagement.	Evaluate and design for affective responses e.g. low-stakes gamification for enjoyment, safe debriefs after simulation to reduce anxiety, interactive apps that build satisfaction and confidence in decision making.

In this review, we have highlighted that there is a clear need for a behavioural gap analysis of current undergraduate curricula to determine how well graduates are being equipped with the capabilities, opportunities and motivations needed for stewardship. Designing interventions that combine cognitive knowledge with practical rehearsal, authentic resources, collaborative teamwork, professional identity development and positive emotional engagement is essential to prepare graduates ready for stewardship practice. Embedding behavioural science principles, such as the COM-B framework, into AMS education will help ensure that future healthcare professionals enter clinical settings with the conditions in place to meet the complex demands of antimicrobial stewardship.

## Supplementary Material

dlaf245_Supplementary_Data

## Data Availability

All data supporting the findings of this study are available within the paper and its Supplementary files.

## Patient and public involvement

Patients and/or the public were not involved in the design, conduct, reporting or dissemination plans of this research.
