# Supplementary material for: A systematic review of antimicrobial stewardship education for undergraduate students in medicine, nursing, pharmacy, dentistry, veterinary science and midwifery using COM-B framework
Source: JAC Antimicrob Resist. 2026 Jan 7;8(1):dlaf245. doi: 10.1093/jacamr/dlaf245 (PMC12776017; doi:10.1093/jacamr/dlaf245)
Supplement: dlaf245_Supplementary_Data [file dlaf245_supplementary_data.docx]

**Table S1.** Full search strategy.

Detailed search queries used across all databases, including controlled vocabulary (e.g., MeSH) and keywords. Filters and logical operators are specified.

**Table S2.** Adapted Medical Education Research Study Quality Instrument (MERSQI) tool for evaluating study quality.

Scoring rubric adapted from the original MERSQI to assess the methodological quality of included educational intervention studies

**Table S3.** The individual rater’s score and agreed score for each Medical Education Research Study Quality Instrument (MERSQI) domain

Scores for each study across all MERSQI domains, showing individual rater assessments and agreed scores used in quality appraisal.

**Table S4.** Coding of interventions in included studies mapped to COM-B domains, with supporting quotes and rationale for absent domains (shaded)

**Table S5.** Agreed domain scores using adapted Medical Education Research Study Quality Instrument (MERSQI)

Final consensus scores across all MERSQI domains for each included study, following independent assessment and reconciliation between two reviewers.

**Table S6.** PRISMA checklist

# Table S1. Full search strategy

| **Database** | **Run number** | **Query** | **Results** |
| --- | --- | --- | --- |
| PUBMED | #1 | "Students, Health Occupations"[MeSH] OR "medical student*" OR "nursing student*" OR "pharmacy student*" OR "dent* student*" OR "veterinary student*" OR "midwifery student*" OR undergraduate OR "healthcare student*" | 205,190 |
|  | #2 | "Antimicrobial Stewardship"[MeSH] OR "antibiotic stewardship" OR "antimicrobial resistance" OR "antibiotic resistance" OR "antimicrobial use" OR antiviral OR antifungal OR antibiotic* OR antimicrobial* | 1,409,919 |
|  | #3 | "Knowledge"[MeSH] OR "Education"[MeSH] OR interprofessional OR learn* OR evaluat* OR assess* OR teach* | 9,854,743 |
|  | #4 | (("Students, Health Occupations"[MeSH] OR "medical student*" OR "nursing student*" OR "pharmacy student*" OR "dent* student*" OR "veterinary student*" OR "midwifery student*" OR undergraduate OR "healthcare student*") AND ("Antimicrobial Stewardship"[MeSH] OR "antibiotic stewardship" OR "antimicrobial resistance" OR "antibiotic resistance" OR "antimicrobial use" OR antiviral OR antifungal OR antibiotic* OR antimicrobial*)) AND ("Knowledge"[MeSH] OR "Education"[MeSH] OR interprofessional OR learn* OR evaluat* OR assess* OR teach*) | 1,016 |
| WEB OF SCIENCE via Clarivate | #1 | TS=("medical student*" OR "nursing student*" OR "pharmacy student*" OR "dent* student*" OR "veterinary student*" OR "midwifery student*" OR undergraduate OR "healthcare student*" ) | 245,651 |
|  | #2 | TS=("Antimicrobial Stewardship" OR "antibiotic stewardship" OR "antimicrobial resistance" OR "antibiotic resistance" OR "antimicrobial use" OR antiviral OR antifungal OR antibiotic* OR antimicrobial*) | 1,041,416 |
|  | #3 | TS=("Knowledge" OR "Education" OR interprofessional OR learn* OR evaluat* OR assess* OR teach*) | 17,088,933 |
|  | #4 | #1 AND #2 AND #3 | 913 |
| APA PsycInfo via Ovid <1806 to January 2025 Week 2> | #1 | ("medical student*" or "nursing student*" or "pharmacy student*" or "dent* student*" or "veterinary student*" or "midwifery student*" or undergraduate or "healthcare student*").mp. [mp=title, abstract, heading word, table of contents, key concepts, original title, tests & measures, mesh word] | 93,543 |
|  | #2 | ("Antimicrobial Stewardship" or "antibiotic stewardship" or "antimicrobial resistance" or "antibiotic resistance" or "antimicrobial use" or antiviral or antifungal or antibiotic* or antimicrobial*).mp. [mp=title, abstract, heading word, table of contents, key concepts, original title, tests & measures, mesh word] | 8,895 |
|  | #3 | ("Knowledge" or "Education" or interprofessional or learn* or evaluat* or assess* or teach*).mp. [mp=title, abstract, heading word, table of contents, key concepts, original title, tests & measures, mesh word] | 26,572,270 |
|  | #4 | #1 AND #2 AND #3 | 41 |
| Embase via Ovid <1974 to 2025 January 21> | #1 | ("medical student*" or "nursing student*" or "pharmacy student*" or "dent* student*" or "veterinary student*" or "midwifery student*" or undergraduate or "healthcare student*").mp. [mp=title, abstract, heading word, table of contents, key concepts, original title, tests & measures, mesh word] | 219,014 |
|  | #2 | ("Antimicrobial Stewardship" or "antibiotic stewardship" or "antimicrobial resistance" or "antibiotic resistance" or "antimicrobial use" or antiviral or antifungal or antibiotic* or antimicrobial*).mp. [mp=title, abstract, heading word, table of contents, key concepts, original title, tests & measures, mesh word] | 1,478,951 |
|  | #3 | ("Knowledge" or "Education" or interprofessional or learn* or evaluat* or assess* or teach*).mp. [mp=title, abstract, heading word, table of contents, key concepts, original title, tests & measures, mesh word] | 14,320,859 |
|  | #4 | #1 AND #2 AND #3 | 2,056 |
| CINAHL Plus via EBSCOhost 1957 - 2025 | #1 | Title “medical student*" OR "nursing student*" OR "pharmacy student*" OR "dent* student*" OR "veterinary student*" OR "midwifery student*" OR undergraduate OR "healthcare student*") | 36,614 |
|  | #2 | Title "Antimicrobial Stewardship" OR "antibiotic stewardship" OR "antimicrobial resistance" OR "antibiotic resistance" OR "antimicrobial use" OR antiviral OR antifungal OR antibiotic* OR antimicrobial*) | 37,190 |
|  | #3 | Title "Knowledge" OR "Education" OR interprofessional OR learn* OR evaluat* OR assess* OR teach*) | 618,174 |
|  | #4 | #1 AND #2 AND #3 | 2,229 |
| SCOPUS | #1 | Article title, Abstract, Keywords “medical student*" OR "nursing student*" OR "pharmacy student*" OR "dent* student*" OR "veterinary student*" OR "midwifery student*" OR undergraduate OR "healthcare student*" | 390,825 |
|  | #2 | Article title, Abstract, Keywords "Antimicrobial Stewardship" OR "antibiotic stewardship" OR "antimicrobial resistance" OR "antibiotic resistance" OR "antimicrobial use" OR antiviral OR antifungal OR antibiotic* OR antimicrobial*) | 1,694,360 |
|  | #3 | Article title, Abstract, Keywords Knowledge OR Education OR interprofessional OR learn* OR evaluat* OR assess* OR teach*) | 23,857,473 |
|  | #4 | #1 AND #2 AND #3 | 1,516 |

# Table S2. Adapted Medical Education Research Study Quality Instrument (MERSQI) tool for evaluating study quality

| Domain | Scoring criteria for each item |
| --- | --- |
| Study  Design | 1.0 = Single-group cross-sectional/posttest  1.5 = Single-group pretest and posttest  2.0 = Non-randomised two-group  3.0 = Randomised controlled trial (RCT) |
| Sampling | Number of institutions  **0.5** = one institution  **1.0** = two institutions  1.5 = three or more institutions  Response rate  0.5 = <50% or not reported  1.0 = 50%-74%  1.5 = ≥75% |
| Type of data | **1.0** = Self-reported surveys only (e.g., perceptions of AMS, confidence ratings)  **2.0** = Mixed subjective and objective assessments (e.g., confidence ratings and knowledge test)  **3.0** = Objective assessments (e.g., OSCEs, prescribing audits, faculty-rated AMS skills) |
| ****Evaluation instrument validity**** | 1.0 = reported (0.0 if absent) Content validity  1.0 = reported (0.0 if absent) Internal structure validity  1.0 = reported (0.0 if absent) Relation with other variables |
| Data analysis | Sophistication of data analysis  1.0 = Descriptive statistics only (e.g., frequencies, means, medians)  2.0 = Inferential statistics (e.g., t-tests, chi-square tests)  3.0 = Multivariate analyses (e.g., regression models)  Appropriateness of data analysis  0.0 = inappropriate statistical analysis or not analysed   1. = appropriate statistical analysis for study design and data type |
| Study outcomes | 1.0 = Self-reported satisfaction, attitudes, perceptions (e.g., post-course surveys on AMS confidence/attitude change)  1.5 = Knowledge/skills assessment using validated tools (e.g., MCQs, OSCEs, or simulation-based prescribing tasks)  2.0 = Performance in faculty-observed AMS decision-making, simulation-based prescribing, or case-based decision-making exercises.  3.0 = Objective performance in AMS decision-making under faculty observation in clinical placements or team-based learning exercises |

*NOTES: This version of the MERSQI was adapted to evaluate the methodological quality of studies included in the review. It includes six domains, Study Design (0–3 points), Sampling (0–3), Type of Data (0–3), Validity of the Evaluation Instrument (0–3), Data Analysis (0–4), and Study Outcomes (0–3), with a maximum total score of 18. The tool was modified to reflect the educational context of antimicrobial stewardship (AMS) interventions for medical and health professions students. Adaptations to the "Study Outcomes" domain reflect the fact that students are not independent prescribers. Therefore, acceptable outcome measures included simulation-based assessments, faculty-observed decision-making exercises, and validated AMS knowledge tests. The "Validity" domain evaluates the presence of content validity evidence, internal structure (e.g., reliability measures), and relationships with other variables (e.g., comparisons between experience levels, or correlations with other instruments). The "Data Analysis" domain considers both the sophistication and appropriateness of statistical techniques used. Detailed scoring criteria for each domain are provided in the table***Table S3. The individual rater’s score and agreed score for each Medical Education Research Study Quality Instrument (MERSQI) domain**

|  | **Study design** | | | **Number of institutions** | | | **Response rate** | | | **Type of data** | | | **Content validity** | | | **Internal structure** | | | **Relationship with variables** | | | **Sophistication of statistics** | | | **Appropriate statistics used** | | | **Study outcomes** | | |
| --- | --- | --- | --- | --- | --- | --- | --- | --- | --- | --- | --- | --- | --- | --- | --- | --- | --- | --- | --- | --- | --- | --- | --- | --- | --- | --- | --- | --- | --- | --- |
| **Study (author, year)** | **1** | **2** | **A*** | **1** | **2** | **A*** | **1** | **2** | **A*** | **1** | **2** | **A*** | **1** | **2** | **A*** | **1** | **2** | **A*** | **1** | **2** | **A*** | **1** | **2** | **A*** | **1** | **2** | **A*** | **1** | **2** | **A*** |
| Aboalshamat *et al.*, 2019 | 3.0 | 3.0 | 3.0 | 0.5 | 0.5 | 0.5 | 0.5 | 1.0 | 1.0 | 2.0 | 2.0 | 2.0 | 0.0 | 0.0 | 0.0 | 0.0 | 0.0 | 0.0 | 0.0 | 0.0 | 0.0 | 2.0 | 2.0 | 2.0 | 1.0 | 0.0 | 1.0 | 1.5 | 1.5 | 1.5 |
| Ahmed *et al.,* 2024 | 1.5 | 1.5 | 1.5 | 0.5 | 0.5 | 0.5 | 0.5 | 0.5 | 0.5 | 2.0 | 2.0 | 2.0 | 1.0 | 0.0 | 1.0 | 1.0 | 0.0 | 1.0 | 0.0 | 0.0 | 0.0 | 2.0 | 2.0 | 2.0 | 1.0 | 1.0 | 1.0 | 1.5 | 1.5 | 1.5 |
| Al Mohajer *et al.,* 2017 | 1.5 | 1.5 | 1.5 | 0.5 | 0.5 | 0.5 | 1.0 | 1.0 | 1.0 | 2.0 | 3.0 | 2.0 | 0.0 | 1.0 | 0.0 | 0.0 | 0.0 | 0.0 | 0.0 | 0.0 | 0.0 | 2.0 | 1.0 | 2.0 | 1.0 | 1.0 | 1.0 | 1.0 | 1.0 | 1.0 |
| Azechi *et al.,* 2022 | 1.5 | 1.5 | 1.5 | 0.5 | 0.5 | 0.5 | 1.0 | 1.0 | 1.0 | 2.0 | 2.0 | 2.0 | 0.0 | 0.0 | 0.0 | 0.0 | 0.0 | 0.0 | 0.0 | 0.0 | 0.0 | 2.0 | 2.0 | 2.0 | 1.0 | 1.0 | 1.0 | 1.5 | 1.5 | 1.5 |
| Badran *et al.,* 2021 | 1.5 | 1.5 | 1.5 | 0.5 | 0.5 | 0.5 | 1.0 | 1.5 | 1.5 | 2.0 | 2.0 | 2.0 | 1.0 | 0.0 | 1.0 | 0.0 | 0.0 | 0.0 | 0.0 | 0.0 | 0.0 | 2.0 | 2.0 | 2.0 | 1.0 | 1.0 | 1.0 | 1.5 | 1.5 | 1.5 |
| Berr *et al.,* 2013 | 1.5 | 1.5 | 1.5 | 1.5 | 1.5 | 1.5 | 0.5 | 0.5 | 0.5 | 2.0 | 1.0 | 2.0 | 0.0 | 0.0 | 0.0 | 0.0 | 0.0 | 0.0 | 0.0 | 0.0 | 0.0 | 2.0 | 1.0 | 2.0 | 1.0 | 1.0 | 1.0 | 1.0 | 1.0 | 1.0 |
| Cerenzio *et al.,* 2021 | 1.5 | 1.5 | 1.5 | 0.5 | 0.5 | 0.5 | 1.5 | 1.5 | 1.5 | 2.0 | 2.0 | 2.0 | 0.0 | 0.0 | 0.0 | 0.0 | 0.0 | 0.0 | 0.0 | 0.0 | 0.0 | 2.0 | 2.0 | 2.0 | 1.0 | 1.0 | 1.0 | 1.5 | 1.5 | 1.5 |
| Cole *et al.,* 2025 | 2.0 | 3.0 | 2.0 | 1.5 | 1.5 | 1.5 | 1.5 | 1.5 | 1.5 | 2.0 | 3.0 | 2.0 | 1.0 | 1.0 | 1.0 | 1.0 | 0.0 | 1.0 | 0.0 | 0.0 | 0.0 | 2.0 | 1.0 | 2.0 | 1.0 | 1.0 | 1.0 | 1.5 | 1.5 | 1.5 |
| Davies *et al.,* 2020 | 1.5 | 1.5 | 1.5 | 0.5 | 0.5 | 0.5 | 1.0 | 1.0 | 1.0 | 2.0 | 2.0 | 2.0 | 0.0 | 0.0 | 0.0 | 0.0 | 0.0 | 0.0 | 0.0 | 0.0 | 0.0 | 1.0 | 1.0 | 1.0 | 1.0 | 1.0 | 1.0 | 1.5 | 1.5 | 1.5 |
| Driesnack *et al.,* 2024 | 1.5 | 1.5 | 1.5 | 0.5 | 0.5 | 0.5 | 1.5 | 1.5 | 1.5 | 2.0 | 2.0 | 2.0 | 1.0 | 0.0 | 1.0 | 0.0 | 0.0 | 0.0 | 0.0 | 0.0 | 0.0 | 2.0 | 2.0 | 2.0 | 1.0 | 1.0 | 1.0 | 1.5 | 1.5 | 1.5 |
| El-Sokkary *et al.,* 2023 | 1.5 | 1.5 | 1.5 | 0.5 | 0.5 | 0.5 | 1.5 | 1.5 | 1.5 | 2.0 | 2.0 | 2.0 | 1.0 | 1.0 | 1.0 | 0.0 | 0.0 | 0.0 | 1.0 | 0.0 | 1.0 | 2.0 | 2.0 | 2.0 | 1.0 | 1.0 | 1.0 | 1.5 | 1.5 | 1.5 |
| Falcione *et al.,* 2014 | 1.5 | 1.5 | 1.5 | 0.5 | 0.5 | 0.5 | 1.0 | 1.0 | 1.0 | 2.0 | 2.0 | 2.0 | 0.0 | 0.0 | 0.0 | 0.0 | 0.0 | 0.0 | 0.0 | 0.0 | 0.0 | 1.0 | 1.0 | 1.0 | 1.0 | 1.0 | 1.0 | 1.5 | 1.5 | 1.5 |
| Gauthier *et al.,* 2015 | 1.5 | 1.0 | 1.5 | 0.5 | 0.5 | 0.5 | 1.5 | 1.5 | 1.5 | 2.0 | 2.0 | 2.0 | 0.0 | 1.0 | 0.0 | 0.0 | 0.0 | 0.0 | 0.0 | 0.0 | 0.0 | 2.0 | 1.0 | 2.0 | 1.0 | 1.0 | 1.0 | 1.5 | 1.5 | 1.5 |
| Guilding *et al.,* 2020 | 1.5 | 1.0 | 1.0 | 1.0 | 1.0 | 1.0 | 1.0 | 1.5 | 1.0 | 2.0 | 1.0 | 1.0 | 0.0 | 1.0 | 1.0 | 0.0 | 0.0 | 0.0 | 0.0 | 1.0 | 1.0 | 2.0 | 2.0 | 2.0 | 1.0 | 1.0 | 1.0 | 1.0 | 1.0 | 1.0 |
| Hidayat *et al.,* 2012 | 1.5 | 1.5 | 1.5 | 0.5 | 0.5 | 0.5 | 1.5 | 1.5 | 1.5 | 1.0 | 2.0 | 1.0 | 0.0 | 1.0 | 0.0 | 0.0 | 0.0 | 0.0 | 0.0 | 0.0 | 0.0 | 2.0 | 2.0 | 2.0 | 1.0 | 1.0 | 1.0 | 1.5 | 1.5 | 1.5 |
| Hussain *et al.,* 2021 | 1.5 | 1.5 | 1.5 | 0.5 | 0.5 | 0.5 | 1.5 | 1.5 | 1.5 | 1.0 | 1.0 | 1.0 | 1.0 | 1.0 | 1.0 | 0.0 | 0.0 | 0.0 | 0.0 | 0.0 | 0.0 | 2.0 | 2.0 | 2.0 | 1.0 | 1.0 | 1.0 | 1.0 | 1.0 | 1.0 |
| Jang *et al.,* 2022 | 2.0 | 2.0 | 2.0 | 0.5 | 1.0 | 1.0 | 1.0 | 1.0 | 1.0 | 1.0 | 1.0 | 1.0 | 0.0 | 0.0 | 0.0 | 0.0 | 0.0 | 0.0 | 0.0 | 0.0 | 0.0 | 2.0 | 2.0 | 2.0 | 1.0 | 1.0 | 1.0 | 1.5 | 1.0 | 1.0 |
| Kufel *et al.,* 2022 | 1.5 | 1.0 | 1.5 | 1.0 | 1.0 | 1.0 | 1.5 | 1.5 | 1.5 | 2.0 | 2.0 | 2.0 | 1.0 | 0.0 | 1.0 | 1.0 | 1.0 | 1.0 | 0.0 | 0.0 | 0.0 | 2.0 | 2.0 | 2.0 | 1.0 | 0.0 | 1.0 | 1.5 | 1.0 | 1.5 |
| Laks *et al.,* 2019 | 1.5 | 1.5 | 1.5 | 0.5 | 0.5 | 0.5 | 1.0 | 1.0 | 1.0 | 2.0 | 2.0 | 2.0 | 0.0 | 0.0 | 0.0 | 0.0 | 0.0 | 0.0 | 0.0 | 0.0 | 0.0 | 2.0 | 2.0 | 2.0 | 1.0 | 1.0 | 1.0 | 1.0 | 1.0 | 1.0 |
| Larnard *et al.,* 2020 | 1.5 | 1.5 | 1.5 | 0.5 | 0.5 | 0.5 | 0.5 | 0.5 | 0.5 | 2.0 | 2.0 | 2.0 | 1.0 | 0.0 | 0.0 | 0.0 | 0.0 | 0.0 | 0.0 | 0.0 | 0.0 | 2.0 | 1.0 | 2.0 | 1.0 | 1.0 | 1.0 | 1.5 | 1.5 | 1.5 |
| Lim *et al.,* 2023 | 1.5 | 1.0 | 1.0 | 1.0 | 1.0 | 1.0 | 1.5 | 1.5 | 1.5 | 3.0 | 3.0 | 3.0 | 1.0 | 1.0 | 1.0 | 0.0 | 1.0 | 0.0 | 0.0 | 0.0 | 0.0 | 1.0 | 1.0 | 1.0 | 1.0 | 0.0 | 1.0 | 1.5 | 1.5 | 1.5 |
| MacCosbe and Segelman, 1984 | 2.0 | 1.5 | 2.0 | 0.5 | 0.5 | 0.5 | 0.5 | 0.5 | 0.5 | 3.0 | 1.0 | 3.0 | 1.0 | 0.0 | 1.0 | 0.0 | 0.0 | 0.0 | 1.0 | 0.0 | 1.0 | 2.0 | 2.0 | 2.0 | 1.0 | 1.0 | 1.0 | 1.5 | 1.0 | 1.5 |
| MacDougall *et al.,* 2017 | 1.5 | 1.5 | 1.5 | 0.5 | 0.5 | 0.5 | 1.5 | 1.5 | 1.5 | 2.0 | 2.0 | 2.0 | 0.0 | 1.0 | 0.0 | 0.0 | 0.0 | 0.0 | 0.0 | 0.0 | 0.0 | 2.0 | 2.0 | 2.0 | 1.0 | 1.0 | 1.0 | 1.5 | 1.5 | 1.5 |
| MacDougall, C., 2017 | 1.5 | 1.0 | 1.5 | 0.5 | 0.5 | 0.5 | 1.5 | 1.5 | 1.5 | 2.0 | 2.0 | 2.0 | 0.0 | 1.0 | 0.0 | 0.0 | 0.0 | 0.0 | 0.0 | 0.0 | 0.0 | 2.0 | 2.0 | 2.0 | 1.0 | 1.0 | 1.0 | 1.5 | 1.5 | 1.5 |
| Malli *et al.,* 2023 | 1.5 | 1.5 | 1.5 | 0.5 | 0.5 | 0.5 | 1.5 | 1.5 | 1.5 | 1.0 | 1.0 | 1.0 | 0.0 | 0.0 | 0.0 | 0.0 | 0.0 | 0.0 | 0.0 | 0.0 | 0.0 | 2.0 | 2.0 | 2.0 | 1.0 | 1.0 | 1.0 | 1.0 | 1.0 | 1.0 |
| Manning *et al.,* 2022 | 1.5 | 1.5 | 1.5 | 0.5 | 0.5 | 0.5 | 1.5 | 1.5 | 1.5 | 1.0 | 1.0 | 1.0 | 0.0 | 0.0 | 0.0 | 0.0 | 0.0 | 0.0 | 0.0 | 0.0 | 0.0 | 1.0 | 1.0 | 1.0 | 1.0 | 1.0 | 1.0 | 1.0 | 1.0 | 1.0 |
| McEwen and Burnett, 2018 | 1.0 | 1.0 | 1.0 | 0.5 | 0.5 | 0.5 | 1.5 | 1.5 | 1.5 | 1.0 | 1.0 | 1.0 | 1.0 | 0.0 | 0.0 | 0.0 | 0.0 | 0.0 | 0.0 | 0.0 | 0.0 | 1.0 | 1.0 | 1.0 | 1.0 | 0.0 | 1.0 | 1.0 | 1.0 | 1.0 |
| McGee *et al.,* 2020 | 1.5 | 1.5 | 1.5 | 0.5 | 0.5 | 0.5 | 1.5 | 1.5 | 1.5 | 1.0 | 1.0 | 1.0 | 0.0 | 1.0 | 1.0 | 0.0 | 0.0 | 0.0 | 0.0 | 0.0 | 0.0 | 2.0 | 1.0 | 2.0 | 1.0 | 0.0 | 1.0 | 1.0 | 1.0 | 1.0 |
| Nori *et al.,* 2017 | 1.5 | 1.5 | 1.5 | 1.0 | 0.5 | 1.0 | 0.5 | 0.5 | 0.5 | 2.0 | 1.0 | 2.0 | 0.0 | 1.0 | 1.0 | 0.0 | 0.0 | 0.0 | 0.0 | 0.0 | 0.0 | 2.0 | 2.0 | 2.0 | 1.0 | 1.0 | 1.0 | 1.0 | 1.0 | 1.0 |
| Nori *et al.,* 2019 | 1.0 | 1.0 | 1.0 | 0.5 | 0.5 | 0.5 | 1.5 | 1.5 | 1.5 | 2.0 | 2.0 | 2.0 | 0.0 | 0.0 | 0.0 | 0.0 | 0.0 | 0.0 | 0.0 | 0.0 | 0.0 | 1.0 | 1.0 | 1.0 | 1.0 | 1.0 | 1.0 | 1.5 | 1.5 | 1.5 |
| Roganović *et al.,* 2024 | 1.5 | 2.0 | 1.5 | 0.5 | 0.5 | 0.5 | 1.5 | 1.5 | 1.5 | 2.0 | 2.0 | 2.0 | 0.0 | 1.0 | 1.0 | 0.0 | 0.0 | 0.0 | 0.0 | 0.0 | 0.0 | 3.0 | 3.0 | 3.0 | 1.0 | 1.0 | 1.0 | 1.5 | 1.5 | 1.5 |
| Rose *et al.,* 2021 | 1.5 | 1.0 | 1.5 | 0.5 | 0.5 | 0.5 | 1.5 | 1.5 | 1.5 | 1.0 | 1.0 | 1.0 | 1.0 | 1.0 | 1.0 | 0.0 | 0.0 | 0.0 | 0.0 | 0.0 | 0.0 | 2.0 | 3.0 | 2.0 | 1.0 | 1.0 | 1.0 | 1.0 | 1.0 | 1.0 |
| Sayyadi-Rahaghi *et al.,* 2023 | 2.0 | 2.0 | 2.0 | 0.5 | 0.5 | 0.5 | 0.5 | 0.5 | 0.0 | 2.0 | 2.0 | 2.0 | 0.0 | 0.0 | 0.0 | 1.0 | 1.0 | 1.0 | 0.0 | 0.0 | 0.0 | 2.0 | 2.0 | 2.0 | 1.0 | 1.0 | 1.0 | 1.5 | 1.5 | 1.5 |
| Sikkens *et al.,* 2018 | 2.0 | 3.0 | 2.0 | 0.5 | 0.5 | 0.5 | 1.5 | 1.0 | 1.5 | 3.0 | 3.0 | 3.0 | 1.0 | 0.0 | 1.0 | 0.0 | 0.0 | 0.0 | 1.0 | 1.0 | 1.0 | 3.0 | 3.0 | 3.0 | 1.0 | 1.0 | 1.0 | 2.0 | 2.0 | 2.0 |
| Stevens *et al.,* 2019 | 1.0 | 1.0 | 1.0 | 0.5 | 0.5 | 0.5 | 0.5 | 0.5 | 0.5 | 2.0 | 1.0 | 2.0 | 0.0 | 0.0 | 0.0 | 0.0 | 0.0 | 0.0 | 0.0 | 0.0 | 0.0 | 1.0 | 1.0 | 1.0 | 1.0 | 0.0 | 1.0 | 1.5 | 1.0 | 1.5 |
| Subasinghe *et al.,* 2024 | 3.0 | 3.0 | 3.0 | 0.5 | 0.5 | 0.5 | 1.5 | 1.5 | 1.5 | 1.0 | 1.0 | 1.0 | 0.0 | 0.0 | 0.0 | 0.0 | 0.0 | 0.0 | 0.0 | 0.0 | 0.0 | 2.0 | 2.0 | 2.0 | 1.0 | 1.0 | 1.0 | 1.0 | 1.0 | 1.0 |
| Sun *et al.,* 2024 | 1.5 | 1.5 | 1.5 | 0.5 | 0.5 | 0.5 | 0.5 | 0.5 | 0.5 | 2.0 | 2.0 | 2.0 | 0.0 | 1.0 | 1.0 | 0.0 | 1.0 | 0.0 | 0.0 | 0.0 | 0.0 | 2.0 | 2.0 | 2.0 | 1.0 | 1.0 | 1.0 | 1.5 | 1.5 | 1.5 |
| Tamboli *et al.,* 2016 | 1.5 | 1.5 | 1.5 | 0.5 | 0.5 | 0.5 | 0.5 | 0.5 | 0.5 | 2.0 | 2.0 | 2.0 | 0.0 | 0.0 | 0.0 | 0.0 | 0.0 | 0.0 | 0.0 | 0.0 | 0.0 | 2.0 | 2.0 | 2.0 | 1.0 | 1.0 | 1.0 | 1.0 | 1.0 | 1.0 |
| Tirupakuzhi *et al.,* 2024 | 1.5 | 1.5 | 1.5 | 1.5 | 1.5 | 1.5 | 1.5 | 1.5 | 1.5 | 2.0 | 2.0 | 2.0 | 1.0 | 1.0 | 1.0 | 0.0 | 0.0 | 0.0 | 0.0 | 0.0 | 0.0 | 2.0 | 2.0 | 2.0 | 1.0 | 1.0 | 1.0 | 1.5 | 1.5 | 1.5 |
| Tulloch *et al.,* 2019 | 1.0 | 1.0 | 1.0 | 0.5 | 0.5 | 0.5 | 1.5 | 1.5 | 1.5 | 1.0 | 1.0 | 1.0 | 0.0 | 0.0 | 0.0 | 0.0 | 0.0 | 0.0 | 0.0 | 0.0 | 0.0 | 1.0 | 1.0 | 1.0 | 1.0 | 1.0 | 1.0 | 1.0 | 1.0 | 1.0 |
| Wang *et al.,* 2021 | 1.0 | 1.0 | 1.0 | 0.5 | 0.5 | 0.5 | 1.5 | 1.5 | 1.5 | 2.0 | 2.0 | 2.0 | 0.0 | 0.0 | 0.0 | 0.0 | 0.0 | 0.0 | 0.0 | 0.0 | 0.0 | 2.0 | 2.0 | 2.0 | 1.0 | 1.0 | 1.0 | 1.5 | 1.5 | 1.5 |
| Yuste *et al.,* 2022 | 1.0 | 2.0 | 1.0 | 0.5 | 0.5 | 0.5 | 1.5 | 1.0 | 1.5 | 2.0 | 2.0 | 2.0 | 0.0 | 0.0 | 0.0 | 0.0 | 0.0 | 0.0 | 0.0 | 1.0 | 0.0 | 2.0 | 2.0 | 2.0 | 1.0 | 1.0 | 1.0 | 1.5 | 1.5 | 1.5 |

*NOTES: 1 = rater 1 score; 2 = rater 2 score and A* = agreed score*

| **Table S4. Coding of interventions in included studies mapped to COM-B domains, with supporting quotes and rationale for absent domains (shaded)** | | | | | | |
| --- | --- | --- | --- | --- | --- | --- |
| **Study (Author, year)** | **Physical Capability** | **Psychological Capability** | **Physical Opportunity** | **Social Opportunity -** | **Reflective Motivation** | **Automatic Motivation** |
| Aboalshamat *et al.,* 2019 | The intervention was purely an online course with questionnaires, involving no hands-on AMS-related procedural skills or physical enactment. | “Section one was composed of 22 questions regarding knowledge of AMR that were either in the form of multiple-choice questions with only one correct answer or questions where participants needed to select all the correct answers from a list.” (Methods, p. 2920) | The intervention provided only online slides and a short video aimed at knowledge improvement. No explicit AMS-enabling resources (e.g., antibiograms, prescribing charts, susceptibility reports) were provided, and students did not actively apply AMS behaviours in a simulated or supervised context. | The intervention consisted of an individual online course with questionnaires but there is no evidence of peer collaboration, interprofessional interaction, expert modelling, or social norms enabling AMS behaviour performance. | The study measured improvements in knowledge scores only; no direct evidence of professional role, identity, goals, self-efficacy, or intentions tied to AMS behaviour. | No explicit emotional, habitual, or reinforcement-based responses to AMS behaviours were reported and outcomes were purely cognitive/knowledge-based. |
| Ahmed *et al.,* 2024 | The intervention consisted of large-group interactive training sessions and questionnaires only, with no evidence of students performing AMS-related procedural skills or hands-on tasks involving physical equipment, patients, or diagnostic tools. | “A structured questionnaire was used to collect baseline data on students’ knowledge regarding antimicrobial resistance and stewardship before conducting training sessions… After ending the training session for each class, post-intervention data was collected using the same questionnaire.” (Methods, p. 3) | Training sessions delivered knowledge and awareness, measured with pre/post questionnaires. No explicit AMS resources (antibiograms, prescribing templates, lab data) were provided and no supervised enactment of stewardship behaviours occurred. | The intervention consisted of large group interactive training sessions and individual pre/post questionnaires to measure AMR/AMS knowledge. Although students were trained together, there is no explicit evidence of peer collaboration, interprofessional teamwork, mentorship, or social norms enabling AMS behaviour. The interaction was focused on knowledge exchange, not performance of AMS tasks. | The study only reports significant increases in AMR and AMS knowledge scores following training sessions with no explicit evidence of students’ role identity, self-efficacy, intentions, goals, or deliberate outcome beliefs linked to AMS behaviours. | No evidence of unconscious emotions, habits, reinforcement, or affective responses tied to AMS behaviour and the outcomes were strictly knowledge-based. |
| Al Mohajer *et al.,* 2017 | The intervention was an interactive online module with case-based MCQs only, there was no enactment of AMS-related procedural skills or physical handling of equipment, specimens, or patients. | “Baseline knowledge was measured via a pretest (12 questions) to assess knowledge about diagnosis and management of common respiratory infections. Improvement of knowledge was assessed via an immediate posttest after finishing the module and a follow-up posttest (at 2 months).” (Methods, p. e15) | Online case-based MCQ knowledge module with no supervised/simulated performance using AMS resources. | The intervention was an individual online module with interactive case simulations and pre/post-tests. Although medical and pharmacy students, as well as residents, all participated, there is no evidence of peer collaboration, interprofessional teamwork, mentorship, or group norms enabling AMS behaviour. The activity was structured as individual completion of cases | The study reported significant improvements in pre-/post-test knowledge scores but did not capture students’ conscious beliefs, professional role identity, self-efficacy, intentions, or goals tied to AMS behaviour. | No evidence of unconscious emotions, reinforcement, or habitual processes tied to AMS behaviours with outcomes were limited to knowledge improvement and retention. |
| Azechi *et al.,* 2022 | The intervention was an on-demand lecture and questionnaire. There is no AMS-related procedural skills or hands-on enactment with equipment, specimens, or patients were described. | “The mean number of correct answers across the eight questions on treatment and diagnosis of infectious diseases and antibiotics was 2.21 ± 1.64 in the prelecture survey… increased to 5.00 ± 1.82 in the postlecture survey (P < 0.001).” (Results, p. 5) | Single on-demand lecture on AMR/AMS and the case examples did not involve active behaviour with provided clinical resources. | The intervention was a single on-demand lecture and individual pre/post questionnaires. Although the lecture included AMS case content (e.g., catheter-related bloodstream infection, common cold), students attended independently online with no peer collaboration, interprofessional teamwork, mentorship, or group norms supporting AMS behaviour. The activity reflects knowledge acquisition only, not AMS task performance in a social context | The study assessed knowledge, attitudes, and perceptions through pre/post-lecture surveys but did not report explicit professional identity, confidence, intentions, or goals linked to AMS behaviours. | No evidence of emotional reactions, habits, or reinforcement tied to AMS behaviour with outcomes focused exclusively on knowledge and perception changes. |
| Badran *et al.,* 2021 | “When the students started their clinical practice… students are allowed to decide on antibiotic prescription for their patients; however, the supervisors interfere in case of inappropriate prescription and give the needed knowledge and training to improve any misconception or irrational prescribing behaviour.” (Discussion, p. 761) | “The students’ knowledge of antibiotics and antibiotic resistance was assessed using a quantitative, structured, self-administered questionnaire… After receiving the educational programme, the students answered the same questionnaire to measure the increase in knowledge from before the educational programme.” (Methods, p. 757 - 758) | “Students designed questionnaires to evaluate the public’s knowledge about antibiotics use and designed different educational materials (posters, leaflets, brochures and presentations), in which they used to educate the patients attending the dental clinics at the university about antibiotics use and antibiotic resistance… A clinical case-based approach was adopted during the lectures to teach students the proper prescribing practices with special emphasis on dental and oral conditions which require antibiotic treatment.” (Methods – Educational Intervention, p. 758–759) | “The sessions were in the form of Lectures, group discussions, small-group interactive role playing sessions during which they were trained to educate the public about antibiotic resistance and the proper use of antibiotics. Students designed questionnaires to evaluate the public’s knowledge about antibiotics use and designed different educational materials (posters, leaflets, brochures and presentations), which they used to educate the patients attending the dental clinics at the university about antibiotics use and antibiotic resistance.” (Methods, p. 758) | “88.9% of the students strongly agreed that the knowledge they have gained about antibiotics helped them when they prescribe it, 95.1% of them share knowledge about antibiotics use and resistance with their patients and/or colleagues, [and] 87.6% stated that the gained knowledge affected their behaviour, practice or skills positively” (Results, p. 759) | Student satisfaction reflects enjoyment of the learning process, not unconscious affective or habitual processes tied to AMS behaviour performance. |
| Berr *et al.,* 2013 | The intervention consisted of an online module (webcast + prescribing game with a virtual patient) and pre/post knowledge questionnaires. No evidence of students performing AMS-related procedural skills (e.g., preparing/administering antibiotics, specimen collection, lab interpretation). Activities were cognitive and online only, not hands-on procedural enactment. | “Online pre and post questionnaires were used to evaluate the impact of the course on the student’s knowledge… The questions concerned the indication when antibiotics should be prescribed, the medical history and prophylaxis as well as the first line regimen of antibiotics used in initial treatment and its dose.” (Materials and Methods, p. e25) | The intervention was limited to an online module (webcast + prescribing game with a virtual patient) and pre/post knowledge questionnaires. Although resources were provided digitally, there is no explicit evidence of supervised or simulated carrying out of AMS behaviours (e.g., prescribing with drug, dose, duration, lab interpretation). This is knowledge-oriented, not opportunity-enabling in a COM-B sense. | The intervention was an online module (Moodle) with a webcast and a virtual prescribing game, completed individually by dental students across three countries. Although students gave feedback on perceived improvement in prescribing skills, there was no evidence of peer collaboration, interprofessional teamwork, mentorship, or group norms enabling AMS behaviour. The activity reflects individual knowledge and skills acquisition only, not AMS task performance in a social context. | The study reports improved questionnaire scores and 70% of students felt their prescribing skills had improved after an online module, but this is framed only as self-reported learning perception and not as explicit reflective beliefs, goals, intentions, or identity statements about enacting AMS behaviours in simulated or supervised contexts. | No direct evidence of affective, habitual, or reinforcement-related drivers explicitly tied to AMS behaviour. The only affect-related finding (“70% felt that their prescribing skills had improved”) refers to perceived skill change, not an emotional or habitual driver during AMS behaviour performance in a simulated or supervised context. |
| Cerenzio *et al.,* 2021 | The workshop/game required students to interpret Gram stains, susceptibilities, select antibiotics, provide dosing, frequency, route, and duration, but this was all done via Google Forms in a case-based interactive game . There is no evidence of hands-on procedural skills with physical equipment, simulated patients, or laboratory enactment. The activity was purely cognitive and digital, not embodied/procedural. | “A patient case was developed… The game was designed to assess knowledge on the ID, interpretation of a Gram stain and antimicrobial susceptibilities… antimicrobial selection, and calculation of antimicrobial stewardship-specific metrics… The final prompt asked for their justification of the antibiotic selection in a short-answer format.” (Methods, p. 1494) | “A patient case was developed involving an individual with hospital-associated pneumonia due to methicillin-resistant S. aureus (MRSA)… The students were required to provide a complete recommendation by entering the dose, route, frequency, and duration… Upon completion of the activity, a group discussion on decision-making rationale and a review of the activity took place.” (Methods – Educational activity and setting, p. 1494–1495) | “Students were randomized into 30 groups of approximately six members, with one leader per group… The activity required students to identify the presented infectious disease, interpret microbiology results, and select antibiotic treatment options. Students were required to provide dosing and antimicrobial stewardship metrics… Upon completion of the activity, a group discussion on decision-making rationale and a review of the activity took place.” (Educational activity and setting, p. 1494) | The study describes students’ enjoyment of a game-based AMS workshop and positive feedback about learning format, but provides no verbatim evidence of reflective beliefs, intentions, goals, or identity tied to performing AMS behaviours (e.g., prescribing, guideline use) in simulated or supervised contexts. | “It was difficult for me to focus on learning because I was feeling stressed or overwhelmed during the workshop” – 49.6% agreed (Table 2, p. 1496). |
| Cole *et al.,* 2025 | The intervention involved written case vignettes and rubrics for evaluating antimicrobial selection and planning, which are cognitive tasks only. No evidence of hands-on procedural enactment with patients, simulators, or diagnostic equipment linked to AMS behaviours was reported. | “The survey also included cases, developed for this study, that were used to assess selection and plan competence. Cases were graded using two study-developed rubrics… The control video… addressed principles of antimicrobial selection. The intervention video… included an explanation of the SODAPOP mnemonic.” (Abstract and Methods, p. 4) | The intervention involved written case vignettes and rubrics for evaluating antimicrobial selection and planning, which are cognitive tasks only. No evidence of hands-on procedural enactment with patients, simulators, or diagnostic equipment linked to AMS behaviours was reported. | The intervention (SODAPOP mnemonic + video with case vignettes) focused on individual reasoning, self-efficacy, and competence in antimicrobial selection. No peer learning, interprofessional interaction, expert modelling, or collaborative AMS decision-making was described as all AMS tasks were completed individually via survey/case responses. | “A statistically significant difference was found in participant-reported self-efficacy pre-scores and post-scores when asked about empiric prescribing (5.8 vs. 6.5; P = .0153) for the SODAPOP group but not the control group.” (Results, p. 4) | No evidence of affective, habitual, or reinforcement processes tied to AMS behaviour as the incentives described were for survey completion, not stewardship behaviour. |
| Davies 2020 | The activity involved playing an antibiotic-themed card game (“Top Trumps”) which required students to recall factual information about antibiotics. There was no evidence of hands-on AMS-related procedural skills (e.g., preparing/administering antibiotics, specimen collection, interpreting cultures hands-on, using PPE). The task remained cognitive/knowledge-based only | “The students were given a standard slide presentation… The students were then asked to complete a short written test… They were then provided with a set of antibiotic Trumps… and then completed a second short written test, containing similar but different questions judged to be of equivalent difficulty.” (Activity, p. 108) | The “Top Trumps” card game involved comparing attributes of antibiotics (spectrum, toxicity, ease of administration) and recall testing. No explicit AMS-enabling resources (e.g., antibiograms, prescribing charts, lab reports) were provided, and students did not enact stewardship behaviours such as drug selection, dosing, or therapy modification in a supervised or simulated context. | The intervention was an antibiotic-themed Top Trumps card game used in pairs for 10 minutes after a lecture. While engaging for learning, it only tested recall of factual content and did not involve peer collaboration, interprofessional teamwork, or supervised carrying out of AMS behaviours such as antibiotic prescribing, stewardship recommendations, or clinical decision-making | The evaluation reports that students found the card game “useful,” “fun,” and a “helpful way to apply knowledge,” but these comments only reflect learning enjoyment and perceptions of teaching efficacy. There are no verbatim student statements showing deliberate beliefs, intentions, goals, identity, or outcome expectancies tied to AMS behaviour being carried out in simulated or supervised settings. | Student comments (e.g., “fun,” “enjoyable”) reflect positive emotions about the learning format but not unconscious or affective drivers tied to performing AMS behaviours. |
| Driesnack *et al.,* 2024 | The study used gamified flashcards, digital games, anonymised microbiological records, and AI-evaluated freeform answers. While these activities developed knowledge and decision-making (Psychological Capability) and gave resources for simulated AMS behaviours (Physical Opportunity), there is no evidence of students performing hands-on procedural skills such as preparing/administering antimicrobials, specimen collection, or manual antibiogram interpretation. | “Our questionnaire contained 18 MC questions about bacteria… antibiotics… and infectious diseases to assess student progress over the course… After the course, significantly more questions were answered correctly… We also designed ten freeform questions containing short use case descriptions… and asked to find a solution… evaluated with AI to reveal learning gaps.” (Methods, p. 2) | “Case studies were also practiced, and students were presented with anonymized, representative microbiological records from real patients. Cases were discussed during in-classroom sessions and in a chat format as homework in the app… The remaining time was aimed at higher-order thinking and acquiring skills in selecting antibiotics for therapy.” (Methods p. 2) | The gamified activity involved students working in small groups on antibiotic “pairs” card decks and discussing clinical cases. While interactive and collaborative, the focus was on skill acquisition and knowledge development (learning behaviours). There is no evidence of supervised AMS behaviour (e.g., prescribing decisions, stewardship recommendations in a clinical or simulated patient context) | “Students felt the course substantially improved their confidence when selecting antibiotics… their assessment changed from 1.61 pre-course … to 3.89 afterward” (Results, p. 3) | The following “Supporting factors of meta-learning include habit formation, which can be achieved through gamified elements…” (Background, p. 2) refers to habit formation through gamification but theoretical and not observed in AMS behaviour. No explicit evidence of affect, reinforcement, or automaticity tied to AMS behaviours was reported. |
| El-Sokkary *et al.,* 2023 | The intervention included interactive sessions, case scenarios, reflective assignments, and video messages, but these did not require students to perform AMS-related procedural skills (e.g., preparing/administering antimicrobials, diagnostic procedures, culture interpretation hands-on). Activities were cognitive/communicative rather than procedural. | “The total knowledge score of the participants has significantly increased after the course compared to the pre-course score; 48.06 ± 6.12 and 52.35 ± 5.91 respectively, p = 0.000… The course was designed with the aim to improve the awareness of medical students about antimicrobial resistance and identify how they could apply the proper prescription rules.” (Results, p. 5) | The elective course provided lectures, interactive sessions, and reflective assignments that improved knowledge, perceptions, and attitudes toward antibiotic resistance, but there is no explicit evidence of AMS-specific resources (e.g., antibiograms, prescribing templates, lab data) or supervised carrying out of prescribing behaviours. All activities remained focused on learning behaviours (discussion, reflection, awareness) rather than performing AMS behaviours. | The elective course combined lectures, interactive sessions, reflective assignments, and group projects (students designed a 2-minute multimedia “health education message”). While students worked in small groups and reflected on interprofessional roles, the activities focused on knowledge, perceptions, and attitudes toward AMR and prescribing. There is no evidence of supervised or collaborative AMS behaviour (e.g., selecting antimicrobials, interpreting antibiograms, stewardship recommendations). | “After the course, students thought they are sufficiently prepared to interpret antibiograms and get the treatment information needed from reliable sources” (Discussion, p. 7) | No explicit evidence of unconscious affect, habit formation, stress/anxiety, or reinforcement tied to AMS behaviours. The changes are framed in terms of reflective beliefs and self-efficacy, not automatic processes. |
| Falcione *et al.,* 2014 | “…4 hands-on stations… designed to elicit recognition of limitations and nuances of common clinical microbiology assays (variation in automated identification and antimicrobial susceptibility, E-test interpretation)… human patient simulation technology… implement empiric treatment and monitoring plans… revise treatment plans…” (Pedagogy/Andragogy, p. 3) | “Learning objectives… were as follows: to correlate physical examination and test data… to implement empiric treatment and monitoring plans… to recognize inaccurate height and weight data… to interpret the significance of measured drug concentrations… to revise treatment plans in response to drug intolerance and in accordance with AMS principles.” (Design, p. 4) | “Groups of 3 to 4 students received the same case scenario, medication profile, microbiological culture, and susceptibility results. The groups were instructed to discuss, propose, and then defend the most appropriate regimen for treatment completion… HPS sessions were conducted in 2 parts over 2 weeks… to implement empiric treatment and monitoring plans, mitigate evolving drug administration-related intolerances, revise treatment plans in response to drug intolerance and in accordance with AMS principles.” (Design – Active Learning Activities / Clinical Cases Using HPS, p. 4) | “Groups of 3 to 4 students received the same case scenario, medication profile, microbiological culture, and susceptibility results. The groups were instructed to discuss, propose, and then defend the most appropriate regimen for treatment completion… HPS sessions were supervised by the course coordinator and facilitated by 2 to 4 pharmacy residents and 2 to 3 ID medicine fellows… designed to mimic hospital patient care rounds.” (Design – Pedagogy/Andragogy & Clinical Cases Using Human Patient Simulation, p. 3) | “Fifty percent of students indicated the simulation cases increased their confidence in the ability to develop a plan for a patient with either BE or CM. All students responded that they strongly agreed or agreed that the simulations helped them develop their ability to solve problems in BE or CM settings” (Evaluation and Assessment, p. 6) | No explicit evidence of affective reactions, reinforcement, habit formation, or unconscious motivational processes tied to AMS behaviours. All reported outcomes focus on confidence and reflective learning, not automatic motivation. |
| Gauthier *et al.,* 2015 | The elective course involved pre-class readings, lectures, question writing, and in-class group case scenario discussions, but there is no evidence of students performing AMS-related procedural skills with physical equipment, specimens, or patients. | “Graded course assessments included completion of preclass assignments… in-class active participation and group presentations, a midpoint examination, and a final examination… This exercise was designed to… engage students in active thinking, problem solving, and reasoning.” (Abstract, p. 1) | Intervention strengthened cognitive skills (knowledge, reasoning, appraisal) but did not meet criteria for physical opportunity. | “Students then were divided at their campuses into small randomly assigned groups of 3 to 4 students. The groups were given 10 to 20 minutes to develop a clinical case scenario in which the AS topic for that week was integrated into a practice setting of their choice. During this time, students designed clinical scenarios to mimic situations in which pharmacists would be expected to implement an AS measure in an institutional setting.” (Design, p. 3) | “I now feel confident that I can make a difference through various AS strategies once I become a pharmacist” (Evaluation and Assessment – Postcourse survey reflections, p. 5) | No evidence of unconscious emotions, habits, or reinforcement processes tied to AMS behaviours. Reported changes were reflective (awareness, understanding, beliefs), not automatic. |
| Guilding *et al.,* 2020 | “Students… complete prescriptions… fill in a hospital prescription chart… calculate the dosage for gentamycin… treatment plan was applied to the SimMan…” (Methods - Table 2, p. 4) | “Students worked together to consider the patients' symptoms and interpret the results of investigations in order to choose the appropriate antimicrobial at each stage of the case and complete prescriptions taking into account dose, duration and route of administration… Students reported acquisition of problem solving and critical evaluation skills, and a wide range of knowledge including concepts and procedures related to infection management and antimicrobial prescribing.” (Abstract and Results, p. 5) | “Students worked together to consider the patients' symptoms and interpret the results of investigations in order to choose the appropriate antimicrobial at each stage of the case and complete prescriptions taking into account dose, duration and route of administration… This required the use of a range of tools, such as the local Antimicrobial Handbook, BNF and the national sepsis management pathway. The collective treatment plan was applied to the SimMan so students could observe the real-time effects of their treatment on the virtual patient.” (Methods - Table 2, p. 4) | “Mixed groups of up to 6 medical and pharmacy students… Students worked together to consider the patients' symptoms and interpret the results of investigations in order to choose the appropriate antimicrobial at each stage of the case and complete prescriptions taking into account dose, duration and route of administration… In the SimMan Sepsis workshop… students respond to a series of clinical questions to try to treat their patient before he deteriorated.” (Methods, Table 2, p. 4) | “Students reflected on their professional roles, identities and responsibilities after participating in all of the workshop sessions… pharmacy students stated… ‘importance of not just working within your own profession/use other professionals to help’… medical students noted the ‘importance of understanding other healthcare professional roles’ and that ‘you need to know everyone’s roles to work efficiently’” (Results, p. 7) | No evidence of unconscious emotions, habits, or reinforcement processes tied to AMS behaviours. Reported changes were reflective (awareness, understanding, beliefs), not automatic. |
| Hidayat *et al.,* 2012 | Students conducted literature review, mini-lectures, journal clubs, and debates based on a paper case but no evidence of performing physical AMS procedures or interacting with patients, simulators, or lab equipment. | “Students were required to critically evaluate the patient case in order to formulate an optimal treatment plan… critically evaluating the infectious-diseases literature and its application in informed clinical judgment… The final active-learning exercise served as a forum for the application of previously acquired knowledge.” (Design, p. 3) | “On the first day of class, students were provided with a patient case along with learning objectives, expectations, and stepwise instructions for the active-learning components and competencies… Students were required to critically evaluate the patient case in order to formulate an optimal treatment plan… During the debate with follow-up discussion, 2 members from each group summarized the advantages and disadvantages of the antimicrobial agent assigned for the patient case, including but not limited to efficacy, toxicity, the potential for development of resistance, dosing feasibility, and cost.” (Design, p. 3–4) | “Students were divided into 4 groups… Each group was assigned a different treatment agent… Students were required to critically evaluate the patient case in order to formulate an optimal treatment plan… The debate with follow-up discussion… was intended to mimic a clinical discussion. Students were expected to apply the knowledge they had gained… to formulate their informed clinical recommendation.” (Design, p. 3) | The outcomes reflects knowledge/awareness (capability), not deliberate beliefs, intentions, or identity tied to AMS behaviour being carried out. | No evidence of unconscious emotions, habits, or reinforcement processes tied to AMS behaviours. Reported changes were reflective (awareness, understanding, beliefs), not automatic. |
| Hussain *et al.,* 2021 | The intervention involved lectures, tutorials, and small group discussions and there was no description of students performing AMS-related procedural skills with physical equipment, specimens, or patients. | “Knowledge of antibiotic stewardship was given to them through lectures, tutorials, small group discussion from May 2019 to December 2020. A structured proforma with questionnaires about antibiotic stewardship was given to them to fill in before and after the learning session.” (Methods, p. 153) | The intervention was limited to lectures, tutorials, and group discussions on AMS. While it improved knowledge and attitudes, there is no evidence of active AMS behaviour enactment with explicit tools or materials. | The intervention was delivered through lectures, tutorials, and small group discussions followed by questionnaires assessing knowledge and attitudes. Although students self-reported improved views on collaboration with interprofessional teams, microbiologists, and pharmacists, there is no evidence of students enacting AMS behaviours (e.g., prescribing decisions, stewardship recommendations, case-based teamwork) in a supervised or peer setting. Therefore, this reflects learning behaviours only, not Social Opportunity. | “After the learning session more than 85% of students significantly agreed that antibiotic stewardship improve patient care, reduce bacterial resistance, decrease hospital stay and cost (p < 0.001).... significant improvement regarding collaborative approaches for the appropriate use of antibiotics, work with inter professional team, role of each profession for appropriate use of antibiotics, collaboration with microbiologist and pharmacist.” (Results, p. 154) | No direct evidence of affective, habitual, or reinforcement-related drivers explicitly tied to AMS behaviour as all findings relate to improved knowledge, attitudes, and intended practices post-session, without description of emotional responses, habit formation, or automatic reinforcement occurring during AMS behaviour performance in a simulated or supervised context. |
| Jang *et al.,* 2022 | “Medical students reviewed the medical records of patients taking antibiotics with a high oral bioavailability and wrote a recommendation for oral conversion after confirmation by an infectious disease specialist.” (Abstract, p. 1) | “Medical students reviewed the medical records of patients taking antibiotics with a high oral bioavailability and wrote a recommendation for oral conversion after confirmation by an infectious disease specialist… A survey on the perception of oral antibiotics was administered to medical students before and after clinical practice to evaluate the educational effect of the program.” (Abstract, p. 1) | “Medical students reviewed the medical records of patients taking antibiotics with a high oral bioavailability and wrote a recommendation for oral conversion after confirmation by an infectious disease specialist.” (Methods, p. 2) | “Medical students reviewed the medical records of patients taking antibiotics with a high oral bioavailability and wrote a recommendation for oral conversion after confirmation by an infectious disease specialist.” (Abstract, p. 1) | The reported outcome (“students were more familiar with which patients could be administered oral antibiotics”) reflects knowledge/capability, not deliberate beliefs, intentions, or identity processes tied to AMS behaviours. | While the study measures increased familiarity and appropriate understanding of criteria for oral conversion after participation, the reported change is a capability gain without explicit emotional, habitual, or reinforcement-related responses during AMS behaviour. No clear affective link beyond knowledge acquisition. |
| Kufel *et al.,* 2022 | “...student-led penicillin allergy counseling interviews, penicillin skin testing simulation, and case studies… pharmacy students performed the scratch component of the penicillin skin test on fellow students as part of this activity.” (Methods, p. 238) | “The pre-intervention survey included 10 knowledge-based PAAST questions including penicillin allergy statistics, penicillin allergy consequences, types of allergic reactions, cross-reactivity between beta-lactam antibiotics, and penicillin allergy management scenarios.” (Methods, p. 238) | “During this session, pharmacy students were exposed to several concepts and areas associated with PAAST… student-led penicillin allergy interviews and activities, a simulated scratch test component of penicillin skin testing, and case studies to assess penicillin allergy scenarios and management strategies… pharmacy students performed the scratch component of the penicillin skin test on fellow students as part of this activity.” (Methods, p. 238) | “The FDIS on PAAST consisted of an infectious disease faculty-led seminar, student-led penicillin allergy counseling interviews, penicillin skin testing simulation, and case studies… Pharmacy students performed the scratch component of the penicillin skin test on fellow students as part of this activity. Finally, pharmacy students worked in groups to complete four graded case studies to evaluate penicillin allergy management strategies with the goal of providing the correct reconciliation strategy and supporting rationale.” (Methods, p. 238) | “Pharmacy students’ PAAST confidence scores (mean±SD) also improved following the interactive instruction and simulation (2.30±0.7 vs 3.22±0.67) with considerable confidence increases in penicillin skin testing” (Abstract, p. 237) | No evidence of unconscious emotional, habitual, or reinforcement processes tied to AMS behaviours. Although students found the class “enjoyable,” affect was linked to learning experience, not AMS performance. |
| Laks *et al.,* 2019 | The intervention was a Moodle-based distance learning course with online simulations, forums, and face-to-face written assessments with no evidence of students performing AMS-related procedural skills with physical equipment, patients, or simulators. | “A written questionnaire… was applied to evaluate participant knowledge before the beginning of the course (‘initial face-to-face assessment’), and… the written questionnaire was again applied (‘final face-to-face assessment’)… In both forms of evaluation, the theoretical concepts of all five modules were addressed, as were aspects related to their clinical application.” (Methods, p. 3) | Online distance-learning provided case-based scenarios and feedback, but did not supply AMS resources (e.g., antibiograms, prescribing templates) for supervised carrying out of stewardship behaviours. | The distance learning course included online simulations, tutor monitoring, and live discussions, but these were focused on knowledge acquisition and assessments. No evidence of AMS task enactment in a social, supervised, or interprofessional context was described | There is a statemtn about valuing AMS as part of the curriculum, not students’ conscious beliefs, identity, or intentions to carry out AMS behaviours in supervised/simulated contexts. | The reported outcomes (knowledge gain, satisfaction, willingness to take another course) reflect learning participation and perceived benefit, not emotional or habitual responses during AMS behaviour performance in a simulated or supervised context. |
| Larnard *et al.,* 2020 | The intervention was a whiteboard animation video series with embedded questions to teach empiric antibiotic selection. All activities were online and cognitive, with no physical AMS-related procedural skills or enactment. | “Students were offered an untimed, nonmandatory 8-question pretest before starting the video series and an untimed, nonmandatory 8-question posttest after completing the video series… Questions were generated by the primary author and reviewed by the other authors who are both faculty in the Division of Infectious Disease.” (Methods, p. 3) | Focused on knowledge building and recall (psychological capability), not provision of physical opportunities for AMS task performance. | The intervention consisted of individually viewed scaffolded video modules on empiric antibiotic selection with embedded questions and tests. While effective for knowledge gain, there was no evidence of peer collaboration, interprofessional teamwork, mentorship, or group norms enabling AMS behaviour enactment. The activity represents learning behaviours only, not social carrying out of AMS tasks. | “After watching this module, I feel more comfortable using the presented antibiotics in the appropriate clinical setting” (Results, p. 3) | No evidence of unconscious emotions, habits, or reinforcement tied to AMS behaviours; enjoyment ratings (“I enjoyed this video module”) reflected learning satisfaction, not AMS behaviours. |
| Lim *et al.,* 2023 | “Station 1 was designed to simulate a hospital setting and involved the student communicating with a simulated doctor. Students were given a drug chart and patient notes to review for any AMS intervention opportunities… Stations 2 and 3… simulate a community/retail pharmacy setting where the student interacted with a simulated patient… Station 2 focussed on prescription counselling of antimicrobials… Station 3 focussed on product requests, prescription problems and customer queries.” (Intervention, p. 647) | “A three-station AMS OSCE was designed and delivered to test the applicability of the interventions set out by WHO’s AMS intervention practical guide… Station 1… involved the student communicating with a simulated doctor… Stations 2 and 3… simulated a community/retail pharmacy setting… This OSCE was a hurdle requirement, meaning students must pass the OSCE to pass the unit.” (Methods, p. 647) | “A three-station AMS OSCE was designed and delivered to test the applicability of the interventions set out by WHO’s AMS intervention practical guide… Station 1 was designed to simulate a hospital setting… Students were given a drug chart and patient notes to review for any AMS intervention opportunities… Station 2 focussed on prescription counselling of antimicrobials… Station 3 focussed on product requests, prescription problems and customer queries.” (Methods , p. 647) | “Station 1 was designed to simulate a hospital setting and involved the student communicating with a simulated doctor. Students were given a drug chart and patient notes to review for any AMS intervention opportunities. Stations 2 and 3 were designed to simulate a community/retail pharmacy setting where the student interacted with a simulated patient. Station 2 focussed on prescription counselling of antimicrobials. Station 3 focussed on product requests, prescription problems and customer queries.” (Methods, p. 647) | “Students were most confident with referral to medical practitioner cases and switching from intravenous to oral or empirical to directed therapy.” (Abstract, p. 646) | Feedback (“OSCE consolidated AMS skills… gave opportunity to exercise principles”) reflects skill practice and opportunity rather than an explicit affective, habitual, or reinforcement driver during AMS tasks. |
| MacCosbe and Segelman 1984 | “The hospital assignment involved the design and implementation of an APR project evaluating empiric antibiotic prescribing by medical residents… students reviewed all orders for antibiotics… recorded on preprinted data sheets… contacted the prescribing residents… recommendations were made to change therapy… follow-up was conducted to determine the outcome of recommendations.” (Methods, p.272) | “The effect of an undergraduate special problems elective upon knowledge of antibiotic therapy was evaluated via a 50-question pre- and posttest… In the elective, students compared antibiotic orders to prescribing guidelines and discussed these orders with the prescribing physicians… recommendations were made to change therapy according to predetermined criteria.” (Abstract, p. 272) | “The hospital assignment involved the design and implementation of an APR project evaluating empiric antibiotic prescribing by medical residents… The prescribing residents were then contacted… In cases where discrepancies could not be justified according to predetermined criteria, recommendations were made to change therapy using the guidelines and follow-up was conducted to determine the outcome of recommendations.” (Methods – Hospital assignment description, p. 272) | “In the elective, students compared antibiotic orders to prescribing guidelines and discussed these orders with the prescribing physicians. When necessary, the students recommended changes in antibiotic therapy according to predetermined criteria.” (Abstract, p. 272) | “In the elective, students compared antibiotic orders to prescribing guidelines and discussed these orders with the prescribing physicians. When necessary, the students recommended changes in antibiotic therapy according to predetermined criteria.” (Methods, p. 272–273) | No evidence of unconscious, affective, or habitual responses tied to AMS behaviours. Reported student activity was deliberate and planned stewardship practice, not automatic motivation. |
| MacDougall *et al.,* 2017 | The curriculum involved online modules and small-group case discussions with branched-logic decision-making. There is no evidence of students performing AMS-related procedural skills with physical equipment, patient simulators, or in clinical settings. | “Students viewed a case vignette online… answered related clinical questions; an online independent learning module on antimicrobial stewardship was subsequently provided; then… students participated in a 2-hour workshop… The cases used a branched-logic format… decisions required in the cases reflected key considerations in antimicrobial stewardship: whether to initiate treatment, impact of patient allergies and drug interactions on antimicrobial selection, adequacy of empiric spectrum of activity, and de-escalation of therapy based on culture results.” (Methods, p. 2) | “In small groups, students revisited Case 1 from the online module and came to a consensus group answer… The small groups then worked through Case 2 together… The decisions required in the cases reflected key considerations in antimicrobial stewardship: whether to initiate treatment, impact of patient allergies and drug interactions on antimicrobial selection, adequacy of empiric spectrum of activity, and de-escalation of therapy based on culture results.” (Methods – Curriculum, p. 2) | “The workshops were led by a physician or pharmacist with expertise in antimicrobial use and consisted of approximately 12 students. Students were subdivided into mixed-professions small groups of 4–5 students. In small groups, students revisited Case 1 from the online module and came to a consensus group answer… The small groups then worked through Case 2 together again followed by group discussion. The cases used a branched-logic format: the decisions students made… reflected key considerations in antimicrobial stewardship: whether to initiate treatment, impact of patient allergies and drug interactions on antimicrobial selection, adequacy of empiric spectrum of activity, and de-escalation of therapy based on culture results.” (Methods, p. 2) | “After completing the curriculum, significantly more students perceived that they were able to describe the role of each profession in appropriate antimicrobial use (34% vs 82%, P < .001), communicate in a manner that engaged the interprofessional team (75% vs 94%, P < .001), and describe collaborative approaches to appropriate antimicrobial use (49% vs 92%, P < .001)” (Results, p. 3) | “Able to describe the role of each profession” is a capability gain. No evidence of affective, habitual, or reinforcement-related drivers during AMS behaviour performance in the cases. |
| MacDougall, C., 2017 | The intervention used active-learning worksheets (“flower diagrams”) for antimicrobial spectrum of activity and in-class exercises, but all activities were cognitive knowledge mapping with no AMS-related procedural skills, physical equipment, patient/simulator interaction, or laboratory behaviours. | “During class, brief concept reviews were followed by active-learning exercises, including a novel schematic method for learning antimicrobial spectrum of activity (‘flower diagrams’)… Students were encouraged to collaborate… practiced learning spectrum of activity using specially designed worksheets… The following quarter… students completed a low-stakes multiple-choice examination on key knowledge from antimicrobial pharmacology.” (Design / Evaluation and Assessment, pp. 2–3) | The intervention focused on active-learning exercises for memorising antimicrobial spectrum of activity using “flower diagrams,” but did not include AMS behaviour enactment (e.g., prescribing, interpreting susceptibility results) in simulated or supervised clinical contexts. | The “flower diagram” innovation was delivered through individual active-learning exercises within lectures. While students could “collaborate with nearby classmates,” the activity was not structured for group decision-making, interprofessional collaboration, mentorship, or cultural norms around AMS behaviours. The focus remained on knowledge retention of antimicrobial spectrum rather than carrying out AMS tasks in a social context. | The study reports improved long-term retention of antimicrobial pharmacology knowledge and positive student feedback (e.g., comments about learning effectiveness and engagement). However, no verbatim student statements reflect deliberate beliefs, intentions, professional identity, or outcome expectancies tied to AMS behaviour enactment. Evidence is limited to knowledge retention and perceptions of teaching method. | The positive sentiment relates to perceived understanding for exam prep. No explicit emotional or reinforcement link to doing AMS behaviours ie the focus is on learning outcomes rather than performing stewardship tasks. |
| Malli *et al.,* 2023 | The intervention was a WHO online antibiotic stewardship course with virtual case scenarios and multiple-choice questions. There is no evidence of students performing AMS-related procedural skills with physical equipment, patient simulators, or in real clinical environments. | “Five case scenarios with two to three multiple-choice questions were used to assess the participant’s knowledge… Pre- and post-course mean knowledge scores were M = 5.36 versus M = 7.66, p < 0.001.” (Results, p. 5) | Online learning provided cognitive knowledge and case-based reasoning practice, but did not provide students with AMS resources (e.g., antibiograms, prescribing templates) to perform behaviours. | The intervention was a WHO-online antibiotic stewardship course delivered virtually to individual students. Outcomes focused on knowledge and confidence change. There is no evidence of peer collaboration, interprofessional teamwork, mentorship, or group norms contributing to AMS behaviour performance. | “After completing the WHO-online antibiotic stewardship course, significantly more students felt confident in prescribing antibiotics, accurately diagnosing infections, selecting appropriate agents, and identifying when antibiotics are not necessary” (Results – Table 2, p. 4) | No evidence of unconscious emotions, habits, or reinforcement processes tied to AMS behaviour as motivational outcomes were expressed as deliberate confidence and capability beliefs. |
| Manning *et al.,* 2022 | The simulation used virtual standardised participants via Zoom. Students engaged in role-played history-taking, discussion, and decision-making but did not perform physical AMS-related procedural skills with actual equipment, specimens, or hands-on patient/simulator interaction in a physical space. | “Qualtrics CoreXM software was used to design the 22-item survey to collect data on students' general antibiotic knowledge (7 items), specific knowledge of the relationship between antibiotic use and development of ABR (8 items), and knowledge of AS nursing practices related to the clinical management of pediatric patients (7 items)… The pre-test launched during the first week of class… The post-test was available immediately following the simulation experience.” (Methods, p. 3) | “For the virtual simulation experience (VSE), students… participated in three, 20-minute sequential interactive SP simulations… Scenarios included: penicillin allergy assessment of an adolescent prior to elective surgery, acute otitis media management of a four-year-old… and urinary tract infection management of a hospitalized four-year-old receiving antibiotics… Students were instructed to approach each SP as they would approach an actual in-person clinical encounter… The faculty member… provided additional information such as vital signs or laboratory results.” (Methods, pp. 2-3) | “Students were randomly pre-assigned to a group of fifteen for a three-hour session. They were further sub-divided into teams of five to participate in three, 20-minute sequential interactive SP simulations… Students were instructed to approach each SP as they would an actual in-person clinical encounter, being attentive to apply appropriate communication, history and physical examination and patient/family teaching skills.” (Methods, p. 3) | This is more about knowledge and skills improvement from simulation, without explicit evidence of deliberate self-efficacy, intentions, or professional identity linked to AMS behaviours. | No evidence of unconscious emotions, habits, or reinforcement processes tied to AMS behaviour. Reported changes were framed as knowledge/skills and deliberate capability, not automatic motivation. |
| McEwen and Burnett 2018 | The AMS teaching used a blended session with presentations, videos, and discussion-based clinical scenarios and although examples of nursing influence in AMS were discussed, there is no evidence of students physically performing AMS-related procedural skills with equipment, specimens, patients, or simulators. | “Integration of acquired theory into mock clinical practice scenarios highlighting associated risk factors and ability to optimise patient safety; promote student engagement, interaction and critical analysis.” (AMS clinical scenario, p. 83) | Session focused on introducing AMS concepts, nursing roles, and scenario discussion, without students performing AMS behaviours using explicit resources. Intervention targeted awareness and cognitive understanding, not provision of physical opportunities for students to practice AMS behaviours. | The AMS education was delivered via lecture + blended teaching (presentation, video, clinical scenarios, and discussion). While scenarios highlighted nursing roles (empiric therapy, sample collection, IPC, patient education), students engaged individually and through discussion only. There is no evidence of peer group enactment, interprofessional teamwork, or supervised AMS behaviour performance (e.g., prescribing decisions, stewardship recommendations). The intervention raised knowledge and awareness, not social enactment of AMS tasks | “The majority of students also stated that they would or have asked questions about a patient’s antibiotic prescription in their placement… Some student nurses stated that they believed that AMS is a contemporary healthcare issue where they have clear roles and responsibilities. Others felt that it made them feel confident about asking questions in their clinical placements about antibiotics and infection prevention practice” (Results – Relevance to pre-registration programme, p. 84) | No evidence of emotions, habits, or reinforcement processes tied to AMS behaviours; reported changes were deliberate intentions and professional identity, not unconscious or affective. |
| McGee *et al.,* 2020 | The structured classroom debate was a cognitive and verbal reasoning exercise using patient cases and literature evidence. The students did not perform AMS-related procedural skills with physical equipment, simulators, or in a clinical setting. | “Given a patient scenario, construct a recommendation based on current guidelines using the core and supplemental strategies of antimicrobial stewardship initiatives… Recommend monitoring parameters given a patient case and be able to defend the interventions.” (Methods p. 221) | “Case vignettes were given to students the day of class. Students were allowed to use a broad range of resources from primary literature to electronic point of care medical references and the class textbook: The Pharmacist’s Guide to Antimicrobial Therapy and Stewardship. The active learning portion of class would always begin with a ‘think-pair-share’ session… Each team discussed the case vignettes and came to an agreement about appropriate interventions based on the evidence from the available resources.” (Methods, p. 221) | “Students were allowed to use a broad range of resources… The active learning portion of class would always begin with a ‘think-pair-share’ session… Students would come together in their groups to build the case. Each team discussed the case vignettes and came to an agreement about appropriate interventions… The lead debaters… presented their group’s plan and interventions and supporting evidence based on the clinical case scenario assigned that week. The debate was modeled to mimic active inpatient medical rounds.” (Methods, p. 221) | “Nineteen students strongly agreed and 11 students agreed that their understanding of the role and functions of an antimicrobial stewardship pharmacist improved at the end of the course. Seventeen students strongly agreed and 11 students agreed that they became confident in justifying the key concepts discussed in the IDSA and SHEA guidelines…” (Findings, p. 225) | No evidence of unconscious habits, affective responses, or reinforcement processes tied to AMS behaviours. Reported outcomes were deliberate confidence and role beliefs, not automatic motivation. |
| Nori *et al.,* 2017 | Although students practiced PPE donning/doffing and hand hygiene with UV tracer (procedural skills), these activities were focused on infection prevention rather than AMS-specific procedural tasks. The AMS elements (e.g., antibiogram use, toolkit building) were delivered through discussion, app use, and MCQs, without direct hands-on enactment of AMS tasks. | “In the first seminar on appropriate antibiotic use, stewardship team members presented fundamental concepts of ‘bug-drug’ matches, de-escalation, and use of the hospital antibiogram/local susceptibility data using case-based multiple-choice questions as reinforcement… Using an audience response system… students answered questions in 3 primary domains: (1) general antibiotic use, (2) principles of microbiology and testing, and (3) prescribing using the local antibiogram.” (Antibiotic Stewardship Seminar – Strategy/Assessment, p. 2) | “In the first seminar on appropriate antibiotic use, stewardship team members presented fundamental concepts of ‘bug-drug’ matches, de-escalation, and use of the hospital antibiogram/local susceptibility data using case-based multiple-choice questions as reinforcement. In small groups, they assembled a ‘toolkit’ of core stewardship strategies to improve antibiotic use in mock clinical scenarios… In year 1 (2014), a printed antibiogram was provided. In years 2 and 3 (2015 and 2016), students downloaded a smartphone application named, ‘APPropriate Use,’ containing antibiograms and clinical practice guidelines developed by Montefiore ASP.” (Methods – Antibiotic Stewardship Seminar, p. 2–3) | “In small groups, they assembled a ‘toolkit’ of core stewardship strategies to improve antibiotic use in mock clinical scenarios.” (Methods, p. 2) | “After app introduction, approximately 70% of students felt comfortable prescribing antibiotics for a known infection compared with 40% at baseline (P = .02)… Approximately 99% agreed that they have a role in promoting patient safety and preventing healthcare-associated infections as medical students” (Results – Preclinical Curriculum: Medical Student Education, Antibiotic Stewardship Seminar & Infection Prevention Seminar, p. 4) | No evidence of emotions, habits, or unconscious responses tied to AMS behaviour as increases in comfort/confidence are reflective, not automatic. |
| Nori *et al.,* 2019 | The seminar used real patient and advocate stories plus expert panel discussion to teach AMS and AMR but all activities were narrative and discussion-based, with no evidence of students performing AMS-related procedural skills with equipment, specimens, patients, or simulators. | “Using an audience-response system, we surveyed students before and after the seminar on their knowledge, attitudes, and perceptions about AS and AMR (primary endpoints). Surveys were designed using a 5-point Likert scale, adapted from previously validated surveys.” (Methods, p. 2) | The seminar consisted of patient and family member storytelling, AMR advocacy discussions, and expert panels; while engaging and potentially motivating, it did not involve explicit AMS-enabling resources for students to perform stewardship tasks in simulated or supervised contexts. | The “Faces of Resistance” seminar exposed students to patient and advocate stories plus expert talks (CDC, IDSA) about AMR and stewardship. While powerful for shaping attitudes and intentions, there is no evidence of students enacting AMS behaviours (e.g., prescribing, case decision-making, stewardship interventions) in a social context. | “Eighty-one percent… agreed that they would modify their future prescribing behaviors… As a medical student and patient advocate, I would like to get involved in stewardship and national efforts to combat drug-resistant infections and C. difficile” (Results – Postseminar survey, Table 1, p. 3) | No evidence of unconscious, affective, or habitual responses tied to AMS behaviours. Reported changes were intentional (reflective), not automatic. |
| Roganović *et al.,* 2024 | The intervention was the use of a mobile application for decision support in antibiotic prescribing but all activities were digital and cognitive, with no evidence of students performing AMS-related procedural skills using physical equipment, specimens, patients, or simulators. | “There was a significant difference in the mean pharmacology test scores between the noAPP and APP groups (5.50 ± 1.80 vs. 7.21 ± 1.03, p = 0.0001)… The major effects of using the app were reflected in the improved inspection of symptoms requiring different management strategies… as well as in the consideration of both symptom severity and patient history.” (Results, p. 4) | “The study involved… practical sessions… with the assistance of a mobile application… The application covers… pulpal and periapical pain and swelling, endocarditis prophylaxis, antibiotic use in pregnancy… conceived based on a decision tree… enabling… quick access to prioritized content… A very useful app and easy to use. Ideal… during the prescribing antibiotics.” (Methods – Development of Mobile Application, p. 7; Results – Feedback on Undergraduate Students’ and Practicing Dentists’ App Experience, p. 3) | The intervention evaluated a mobile application for dental antibiotic prescribing used by students and practicing dentists. While students reported it was useful for learning and decision-making, all tasks were completed individually via the app, with no evidence of peer collaboration, interprofessional teamwork, mentorship, or group norms enabling AMS behaviour to be carried out. | “A very useful app and easy to use. Ideal as a reminder for studying, and also during the prescribing antibiotics.” (Results – Feedback, p. 2) | “Simple, practical app, I am satisfied.” (Results – Feedback, p. 2) |
| Rose *et al.,* 2021 | The active learning session involved group completion of antibiotic charts and case-based “bugs and drugs” identification games but all activities were cognitive and discussion-based, with no evidence of students performing AMS-related procedural skills with physical equipment, patient simulators, or in real clinical environments. | “The learning objectives for our session… were for students to 1) differentiate the major antibiotic classes including mechanism of action and major side effects and 2) demonstrate knowledge of appropriate antibiotic utilization for commonly encountered infections… In the second segment, the students reviewed seven clinical cases to highlight common infectious conditions and appropriate antibiotic utilization.” (Methods, p. 2) | Students were asked to list the relevant “bugs and drugs” for each case. They then shared rationale with peers, and faculty corrected or added commentary. This is primarily knowledge recall & categorisation (pathogen–antibiotic matching), not full prescribing/stewardship behaviours. There’s no mention of dose, frequency, duration, route, susceptibility interpretation, de-escalation, or stewardship recommendations. The “chart completion” is also knowledge -structuring, not carrying out AMS tasks | “In the second segment, the students reviewed seven clinical cases to highlight common infectious conditions and appropriate antibiotic utilization… students worked in small groups (5–10 students) to list the relevant ‘bugs and drugs’ on an index card… The first group… was asked to describe their answers and rationale to the rest of the students. The facilitators helped to highlight important teaching points or provide additional commentary, including correcting any inaccuracies in the student presentations.” (Methods, pp. 2–3) | The study reports student enjoyment of the active learning format and perceived improvements in understanding antibiotic use (e.g., “this lecture improved my understanding of antibiotics and their appropriate clinical use”) but provides no verbatim student statements reflecting deliberate beliefs, professional identity, intentions, or outcome expectancies tied to AMS behaviour in supervised/simulated practice. | Although the exercise involved AMS behaviour (case-based bug/drug matching), the affective comment (“a lot of fun”) is linked to the game format, not directly to the act of AMS behaviour performance therefore fails behavioural salience check under strict criteria. |
| Sayyadi-Rahaghi *et al.,* 2023 | The intervention was an E-learning course with online video lectures and multiple-choice examinations; no evidence of students performing AMS-related procedural skills with physical equipment, patient simulators, or in real clinical environments. | “After the end of E-learning and face-to-face courses, a similar examination with 19 questions was used including four dimensions of selecting the appropriate antibiotics, understanding the side effects of in AAP, familiarity with the types of antibiotics and their mechanism of action, and understanding the spectrum of effect and clinical application of antibiotics from both groups.” (Materials and Methods, p. 15) | The intervention was an online E-learning course covering appropriate antibiotic prescribing content, but there is no explicit description of students using AMS-enabling resources (e.g., antibiograms, patient data, prescribing charts) to perform AMS behaviours in simulated or supervised contexts. | The intervention was a quasi-experimental e-learning course (videos + online LMS delivery) compared with face-to-face lectures. Outcomes measured were knowledge scores and satisfaction, with no mention of peer collaboration, interprofessional teamwork, mentorship, or supervised AMS task enactment (e.g., prescribing, counselling, culture interpretation). | The study assessed the impact of e-learning on students’ knowledge of appropriate antibiotic prescribing and measured satisfaction with the teaching format. While improvements in understanding and satisfaction were reported, no verbatim evidence was provided of students’ reflective beliefs, intentions, professional identity, or outcome expectancies explicitly tied to performing AMS behaviours in simulated or supervised contexts. | The study reports improved knowledge and medium satisfaction with the E-learning course, but there is no direct evidence of affective, habitual, or reinforcement-related drivers explicitly tied to AMS behaviour. Satisfaction statements relate to the educational method, not to emotions or habits during performance of AMS behaviours in a simulated or supervised context. |
| Sikkens *et al.,* 2018 | “Six months later, all students underwent an infectious disease-based objective structured clinical examination (OSCE) aimed at simulating postgraduate prescribing… set up… including use of a patient actor… The final product… was a written prescription for an infectious disease case.” (Methods, p. 2244) | “Six months later, all students underwent an infectious disease-based objective structured clinical examination (OSCE) aimed at simulating postgraduate prescribing… Main outcomes were the OSCE pass percentage and knowledge, drug choice and overall scores… The antimicrobial knowledge tests comprised 57 multiple-choice questions each, validated by several experts.” (Abstract and Methods, p. 2243) | “The objective structured clinical examination (OSCE) aimed to simulate prescribing behaviour in clinical practice… The final product of the exam was a written prescription for an infectious disease case. Students were scored on overall performance based on a standardized score system including subscores for drug choice and knowledge.” (Methods – Measurements, p. 2244) | The intervention was an individual e-learning module on antimicrobial prescribing, followed by an OSCE with a patient actor to assess competence. While the OSCE simulated prescribing, it was conducted individually and there is no evidence of peer collaboration, interprofessional teamwork, or supervised social performance of AMS behaviours during the intervention. | “When questioned on their confidence in prescribing antimicrobial therapy in clinical practice prior to and subsequent to the e-learning, the percentage of students indicating insecurity or severe insecurity decreased from 74% to 37% (P = 0.002)” (Results, p. 2244) | No explicit evidence of emotional reactions, reinforcement, or habits tied to performing AMS behaviours was reported. Confidence gains are reflective rather than automatic. |
| Stevens *et al.,* 2019 | The intervention was an online case-based learning resource with quizzes on antibiotic prescribing but all activities were digital and cognitive, with no evidence of students performing AMS-related procedural skills using physical equipment, patients, or simulators. | “The module consisted of the six interactive tutorials with accompanying quizzes and feedback… Five interactive online case-based tutorials… Students had to choose an appropriate empirical antibiotic… and rationalize empirical choice to an appropriate and more directed antibiotic… To progress through the module, students had to obtain a goal grade of ≥90% in each quiz before accessing the next online activity.” (Design of the online module, p. 2) | “Five interactive online case-based tutorials… included: (i) Clostridioides difficile infection, (ii) pyelonephritis, (iii) cellulitis, (iv) septic arthritis and (v) bacterial meningitis… Relevant clinical information was then provided to the students… Students had to choose an appropriate empirical antibiotic… more information, such as laboratory results and susceptibility testing results, [was] provided. On this basis, the students were asked to rationalize their treatment.” (Methods – Design of the online module, p. 2–3) | The intervention was an online case-based module with interactive tutorials and quizzes where students individually selected antibiotics, interpreted labs, and rationalised therapy. While this involved AMS-related decisions, all activities were completed individually through e-learning, with no peer collaboration, interprofessional teamwork, mentorship, or group norms enabling AMS behaviour to be performed. | “Only 33% (13/39) reported the resource would change their practice” (Results, p. 5) | Although the module involved AMS behaviour (case-based antibiotic selection and rationalisation), the student feedback in the paper only refers to general satisfaction, relevance, and perceived quality. No direct emotional, habitual, or reinforcement-related responses tied specifically to performing AMS behaviours were reported. |
| Subasinghe *et al.,* 2024 | The AMRSim intervention was an online, virtual scenario-based simulation with note-taking in a workbook and group discussion with no evidence of students performing AMS-related procedural skills with actual equipment, physical patient simulators, or real-life enactment. | “Significant differences between groups were found for outcomes measures related to knowledge about… sources and spread of infection in veterinary practice, HH, AMR, the relationship between IPC and AMR, the role of IPC in AMS, and knowledge and confidence about the role of PPE and disinfection use for IPC. In all of these cases, the IG had significantly higher mean scores than the WLCG.” (Results, journal p. 56) | “The novel digital teaching tool used for the intervention, AMRSim, is an interactive, 3-Dimensional… simulator of a veterinary practice within which humans, animals, and bacteria interact, and contamination is transferred… mimics a real-life veterinary clinical practice scenario: a dog being prepared for a hind limb surgical procedure… Participants… completed all experimental tasks individually… an online workbook was used… to make notes.” (Methods – The Intervention (Antimicrobial Reality Simulator: AMRSim and Workshop Transcript), p. 3–4) | “The students indicated that immediate feedback from the facilitator and open peer discussion helped their learning: ‘I really liked the discussion we had as a group, it was very helpful and confirmed/solidified my knowledge around this topic.’ Peer learning was commonly cited as beneficial, providing ‘ideas from other people that I would have missed.’” (Results – Student Feedback, p. 4 of the PDF) | “Student feedback commended the visual representation of the clinical scenario… The intervention also stimulated self-reflection with most students stating that they would be more aware of their own IPC practice and role in AMS in future placements, indicating a motivation to change behaviour: ‘before the workshop I was more inclined to use antibiotics but now I realise that was foolish’” (Results – Student Feedback, Table 2, p. 14) | No explicit evidence of emotions, reinforcement, or habit formation tied to AMS behaviours (IPC enjoyment excluded). |
| Sun *et al.,* 2024 | The study was an online survey measuring veterinary students’ knowledge, confidence, and perceptions before and after clinical rotations and while clinical cases were discussed, there is no evidence of students performing AMS-related procedural skills with equipment, specimens, patients, or simulators. | “Nine clinical AMU scenarios were used to test respondents’ clinical AMU knowledge, understanding of guidelines for empiric AMU, and interpretation of sensitivity testing results.” (Survey Design and Pilot, journal p. 370) | Assessed students’ cognitive knowledge of AMS principles, but did not provide physical opportunities to perform prescribing/stewardship tasks using resources. | The intervention was a survey study measuring veterinary students’ knowledge, confidence, and awareness of AMS before and after clinical rotations. Although clinical exposure was discussed, the study reports only on knowledge/attitude changes.There is no evidence of structured peer collaboration, interprofessional teamwork, mentorship, or group enactment of AMS behaviours during the intervention. | The study measured veterinary students’ knowledge and confidence scores pre- and post-clinical rotations, but both remained low with no significant change. While it highlights gaps in AMS preparedness and awareness of guidelines, the text does not include verbatim evidence of reflective beliefs, professional identity, intentions, goals, or outcome expectancies tied to AMS behaviour enactment in supervised/simulated settings. | Although the study measures changes in self-reported confidence before and after clinical rotations, there is no explicit description of emotional, habitual, or reinforcement-related drivers tied to performing AMS behaviours in a simulated or supervised context. Confidence measures are aggregated survey scores without qualitative evidence linking affect directly to AMS behaviour. |
| Tamboli *et al.,* 2016 | The intervention was a 2-hour educational session on antimicrobial use and resistance followed by questionnaire completion and no evidence of students performing AMS-related procedural skills with physical equipment, patients, or simulators. | “A modified questionnaire… consisted of 9 questions of knowledge of antimicrobial prescribing and awareness about antimicrobial resistance… The participants were made to undergo a training session of 2 hours regarding the empirical and rational use of antimicrobials, preventing/avoiding antimicrobial resistance… After the training session, the participants were asked again to answer the same questionnaire administered to them and the response was documented.” (Methods, journal p. 1545) | The intervention was a 2-hour educational session on empirical and rational antimicrobial use and AMR prevention, assessed via a pre/post questionnaire. There is no evidence of students using AMS-enabling resources (e.g., patient cases, lab data, guidelines) to do any AMS behaviours in a simulated or supervised context. | The intervention was a 2-hour didactic educational session followed by questionnaires measuring knowledge and attitudes among medical, dental, and nursing students. While it highlighted awareness and attitudes, there is no evidence of peer collaboration, interprofessional teamwork, or supervised enactment of AMS behaviours (e.g., prescribing, case review, stewardship recommendations). | The study measured knowledge and attitude scores before and after an educational session, showing statistically significant improvements. However, the data are presented as questionnaire scores and percentages (e.g., awareness of AMR, belief in abuse of antimicrobials, desire for more education) rather than verbatim reflective statements. There is no direct evidence of students’ beliefs, professional identity, intentions, or outcome expectancies tied to performing AMS behaviours in supervised or simulated contexts. | Although the study reports statistically significant improvement in knowledge and attitude scores after the session, no qualitative or quantitative evidence describes emotional, habitual, or reinforcement-related drivers explicitly tied to performing AMS behaviours in a simulated or supervised context. All reported outcomes relate to knowledge gain and general attitudes toward antimicrobial use/resistance, not to affect during carrying out of stewardship behaviours. |
| Tirupakuzhi *et al.,* 2024 | The intervention was an entirely online educational program with pre- and post-tests. There is no evidence of students performing AMS-related procedural skills, handling equipment, or engaging in hands-on simulations. Activities were purely cognitive/knowledge-based, so it does not meet the Physical Capability inclusion criteria. | “Students were then provided access to online educational material comprising 20 short lectures… covered a range of topics which included basics of microbiology, fundamental concepts in AMR and stewardship, diagnosis and management of common infections, basics of antimicrobial pharmacokinetics and pharmacodynamics, and vaccination” (Methods, journal p. 2) | The intervention was an online lecture-based educational intervention to increase AMR knowledge. No provision of AMS-enabling resources for behaviour enactment. | The educational intervention targeted knowledge acquisition and awareness (psychological capability), but did not create or alter the social environment supporting AMS behaviours and therefore not social opportunity. | The study reports only knowledge improvements and general professional sentiments. No verbatim evidence of beliefs, intentions, goals, or identity tied to AMS behaviour enactment in supervised or simulated contexts. | The study reports only quantitative improvements in knowledge test scores and discusses curriculum changes and role-modelling in a general sense. There is no qualitative or quantitative evidence of affective, habitual, or reinforcement-related drivers explicitly tied to the performing of AMS behaviours in a simulated or supervised context. All measured outcomes relate to cognitive learning gains rather than motivation during behaviour. |
| Tolloch *et al.,* 2019 | The modified TBL intervention involved pre-class readings/webcasts, readiness assurance tests, and in-class team case discussions with multiple-choice application questions. All activities were cognitive and discussion-based, with no evidence of students performing AMS-related procedural skills using physical equipment, patients, or simulators. | “In-class individual and team readiness assurance testing (IRAT and TRAT, respectively) consisted of a ten-question multiple-choice quiz… Lastly, for the application activity, students were guided through a real-life clinical scenario punctuated by a series of multiple-choice questions, which mirrored real clinical conundrums… Groups of students… were encouraged to defend their answers and ask clarifying questions of the supervising faculty.” (Intervention, p. 1180) | Students worked through clinical scenarios with MCQs and group defense. This is structured reasoning/peer discussion, but no explicit provision of AMS resources (e.g., charts, antibiograms). | “The antimicrobial stewardship course consisted of two 120-min modules… randomly selected groups of six to eight students… In-class individual and team readiness assurance testing… The answers… were discussed after the TRAT. Lastly, for the application activity, students were guided through a real-life clinical scenario punctuated by a series of multiple-choice questions… Groups of students had to simultaneously reveal the answer… and were encouraged to defend their answers and ask clarifying questions of the supervising faculty.” (Methods – Intervention, pp. 1180) | “I could appreciate that the discussions we were having are the kind of discussions that occur every day in the hospital for patient care and antimicrobial stewardship. This required critically thinking and it was refreshing.” (Table 1, p. 1182) | The word “refreshing” is a retrospective appraisal of the learning experience, not an in-the-moment emotional reaction during AMS behaviour. No evidence of emotion, habit, or reinforcement tied directly to stewardship actions. |
| Wang *et al.,* 2021 | “Students performed prospective audit-and-feedback activities… participated in daily AS rounds… suggested and implemented interventions in real time to frontline providers… reviewed patients undergoing therapeutic drug monitoring for vancomycin and recommended dosing changes under the supervision of an ID pharmacist.” (Curriculum, p.2) | “Students participated in small-group didactic sessions reviewing the pharmacokinetics and spectra of activity of commonly used antibiotics… were provided with clinical cases to practice applying this framework… students completed case-based modules reviewing common facets of AS in everyday practice… participated in daily AS rounds… suggested and implemented interventions in real time… reviewed patients undergoing therapeutic drug monitoring for vancomycin and recommended dosing changes under the supervision of an ID pharmacist.” (Curriculum, pp. 1–3) | “Students performed prospective audit-and-feedback activities at the Hospital of the University of Pennsylvania, with daily assignments to review electronic stewardship alerts… participated in daily AS rounds with ID physicians, pharmacists, and other interprofessional… trainees, during which they suggested and implemented interventions in real time… reviewed patients undergoing therapeutic drug monitoring for vancomycin and recommended dosing changes under the supervision of an ID pharmacist.” (Methods – Curriculum, p. 3) | “Students performed prospective audit-and-feedback activities at the Hospital of the University of Pennsylvania, with daily assignments to review electronic stewardship alerts. Subsequently, they participated in daily AS rounds with ID physicians, pharmacists, and other interprofessional… trainees, during which they suggested and implemented interventions in real time to frontline providers using the ‘3Ps, 3Ds, and 3Cs’ AS framework. Last, to enhance their confidence… students reviewed patients undergoing therapeutic drug monitoring for vancomycin and recommended dosing changes under the supervision of an ID pharmacist.” (Methods – Curriculum, p. 2) | “By course completion, confidence increased to 100% in each domain [antibiotic selection, dosing for agents requiring therapeutic drug monitoring, and adverse effects]” (Results – Table 2, p. 3) | No explicit evidence of affective reactions, habit formation, reinforcement, or unconscious motivational processes tied to AMS behaviours. All outcomes focus on reflective confidence and learning. |
| Yuste *et al.,* 2022 | The study was a cross-sectional survey evaluating students’ knowledge, perceptions, and attitudes before and after infectious diseases training. All activities were survey completion and classroom teaching so there is no evidence of students performing AMS-related procedural skills with physical equipment, patient simulators, or in real clinical settings. | “Knowledge of antibiotic stewardship underwent a statistically significant change after training in infectious diseases (from 9.2% in group 1 to 52.2% in group 3, p < 0.001)… In the training questions block we also found an increase in the average number of correct answers (21.4% in group 1 vs 44.7% in group 3, p < 0.001).” (Results, journal p. 4) | The intervention was limited to infectious diseases lectures and a survey assessing knowledge, attitudes, and perceptions; there is no evidence of students using AMS-enabling resources (e.g., patient cases, lab data, prescribing tools) to enact stewardship behaviours in simulated or supervised contexts. | The study was a cross-sectional survey of Spanish medical students at different stages of infectious diseases training. Outcomes measured = knowledge, perceptions, and attitudes toward AMR and antibiotic use. There is no evidence of peer collaboration, interprofessional teamwork, mentorship, or group enactment of AMS behaviours (e.g., prescribing, de-escalation, stewardship recommendations) in the educational activities described. | “98.4% think the problem of antibiotic resistance should be considered before starting an antibiotic prescription.”; “90.3% of them believed that prior microbiological sample collection always or frequently should be performed.”: “84.2% of fully trained students always or frequently considered conversion from parenteral to oral treatment when available.”; “Most of the students with complete training considered prolonged therapy unnecessary.”; “After training in infectious diseases, knowledge of the stewardship antibiotic program increases from 9.3%... to 52.2%... This situation contributes to a better perception of the problem of antibiotic resistance and a better and more rational use of these agents.” (Results – Students’ knowledge, p. 2-5 (Table 2 and text) and Discussion, p. 6) | The study reports improvements in knowledge, awareness, and attitudes after infectious diseases training, but there is no direct evidence of emotional, habitual, or reinforcement-related drivers explicitly tied to performing AMS behaviours in a simulated or supervised context. Reported perceptions (e.g., viewing AMR as a public health problem, considering it before prescribing) are cognitive/reflective, not affective or automatic motivational responses during performance of AMS tasks. |

# Table S5. Agreed domain scores using adapted Medical Education Research Study Quality Instrument (MERSQI).

| **Study**  **(Author, Year)** | **Study Design**  **(0–3)** | **Sampling**  **(0–3)** | **Type of Data**  **(0–3)** | **Validity**  **(0–3)** | **Data Analysis**  **(0–4)** | **Study Outcomes**  **(0–3)** | **Total MERSQI Score (Max = 18)** |
| --- | --- | --- | --- | --- | --- | --- | --- |
| Aboalshamat *et al.,* 2019 | 3.0 | 1.5 | 2.0 | 0.0 | 3.0 | 1.5 | 11.0 |
| Ahmed *et al.,* 2024 | 1.5 | 1.0 | 2.0 | 2.0 | 3.0 | 1.5 | 11.0 |
| Al Mohajer *et al.,* 2017 | 1.5 | 1.5 | 2.0 | 0.0 | 3.0 | 1.0 | 9.0 |
| Azechi *et al.,* 2022 | 1.5 | 1.5 | 2.0 | 0.0 | 3.0 | 1.5 | 9.5 |
| Badran *et al.,* 2021 | 1.5 | 2.0 | 2.0 | 1.0 | 3.0 | 1.5 | 11.0 |
| Berr *et al.,* 2013 | 1.5 | 2.0 | 2.0 | 0.0 | 3.0 | 1.0 | 9.5 |
| Cerenzio *et al.,* 2021 | 1.5 | 2.0 | 2.0 | 0.0 | 3.0 | 1.5 | 10.0 |
| **Cole *et al.,* 2025** | **2.0** | **3.0** | **2.0** | **2.0** | **3.0** | **1.5** | **13.5** |
| Davies *et al.,* 2020 | 1.5 | 1.5 | 2.0 | 0.0 | 2.0 | 1.5 | 8.5 |
| Driesnack *et al.,* 2024 | 1.5 | 2.0 | 2.0 | 1.0 | 3.0 | 1.5 | 11.0 |
| **El-Sokkary *et al.,* 2023** | **1.5** | **2.0** | **2.0** | **2.0** | **3.0** | **1.5** | **12.0** |
| Falcione *et al.,* 2014 | 1.5 | 1.5 | 2.0 | 0.0 | 2.0 | 1.5 | 8.5 |
| Gauthier *et al.,* 2015 | 1.5 | 2.0 | 2.0 | 0.0 | 3.0 | 1.5 | 10.0 |
| Guilding *et al.,* 2020 | 1.0 | 2.0 | 1.0 | 2.0 | 3.0 | 1.0 | 10.0 |
| Hidayat *et al.,* 2012 | 1.5 | 2.0 | 1.0 | 0.0 | 3.0 | 1.5 | 9.0 |
| Hussain *et al.,* 2021 | 1.5 | 2.0 | 1.0 | 1.0 | 3.0 | 1.0 | 9.5 |
| Jang *et al.,* 2022 | 2.0 | 2.0 | 1.0 | 0.0 | 3.0 | 1.0 | 9.0 |
| **Kufel *et al.,* 2022** | **1.5** | **2.5** | **2.0** | **2.0** | **3.0** | **1.5** | **12.5** |
| Laks *et al.,* 2019 | 1.5 | 1.5 | 2.0 | 0.0 | 3.0 | 1.0 | 9.0 |
| Larnard *et al.,* 2020 | 1.5 | 1.0 | 2.0 | 0.0 | 3.0 | 1.5 | 9.0 |
| Lim *et al.,* 2023 | 1.0 | 2.5 | 3.0 | 1.0 | 2.0 | 1.5 | 11.0 |
| **MacCosbe and Segelman, 1984** | **2.0** | **1.0** | **3.0** | **2.0** | **3.0** | **1.5** | **12.5** |
| MacDougall *et al.,* 2017 | 1.5 | 2.0 | 2.0 | 0.0 | 3.0 | 1.5 | 10.0 |
| MacDougall, C., 2017 | 1.5 | 2.0 | 2.0 | 0.0 | 3.0 | 1.5 | 10.0 |
| Malli *et al.,* 2023 | 1.5 | 2.0 | 1.0 | 0.0 | 3.0 | 1.0 | 8.5 |
| Manning *et al.,* 2022 | 1.5 | 2.0 | 1.0 | 1.0 | 2.0 | 1.0 | 8.5 |
| McEwen and Burnett, 2018 | 1.0 | 2.0 | 1.0 | 0.0 | 2.0 | 1.0 | 7.0 |
| McGee *et al.,* 2020 | 1.5 | 2.0 | 1.0 | 1.0 | 3.0 | 1.0 | 9.5 |
| Nori *et al.,* 2017 | 1.5 | 1.5 | 2.0 | 1.0 | 3.0 | 1.0 | 10.0 |
| Nori *et al.,* 2019 | 1.0 | 2.0 | 2.0 | 0.0 | 2.0 | 1.5 | 8.5 |
| **Roganović *et al.,* 2024** | **1.5** | **2.0** | **2.0** | **1.0** | **4.0** | **1.5** | **12.0** |
| Rose *et al.,* 2021 | 1.5 | 2.0 | 1.0 | 1.0 | 3.0 | 1.0 | 9.5 |
| Sayyadi-Rahaghi *et al.,* 2023 | 2.0 | 0.5 | 2.0 | 1.0 | 3.0 | 1.5 | 10.0 |
| Sikkens *et al.,* 2018 | 2.0 | 2.0 | 3.0 | 2.0 | 4.0 | 2.0 | 15.0 |
| Stevens *et al.,* 2019 | 1.0 | 1.0 | 2.0 | 0.0 | 2.0 | 1.5 | 7.5 |
| Subasinghe *et al.,* 2024 | 3.0 | 2.0 | 1.0 | 0.0 | 3.0 | 1.0 | 10.0 |
| Sun *et al.,* 2024 | 1.5 | 1.0 | 2.0 | 1.0 | 3.0 | 1.5 | 10.0 |
| Tamboli *et al.,* 2016 | 1.5 | 1.0 | 2.0 | 0.0 | 3.0 | 1.0 | 8.5 |
| **Tirupakuzhi *et al.,* 2024** | **1.5** | **3.0** | **2.0** | **1.0** | **3.0** | **1.5** | **12.0** |
| Tulloch *et al.,* 2019 | 1.0 | 2.0 | 1.0 | 0.0 | 2.0 | 1.0 | 7.0 |
| Wang *et al.,* 2021 | 1.0 | 2.0 | 2.0 | 0.0 | 3.0 | 1.5 | 9.5 |
| Yuste *et al.,* 2022 | 1.0 | 2.0 | 2.0 | 0.0 | 3.0 | 1.5 | 9.5 |
|  |  |  |  |  |  |  |  |

*Notes: All domain scores represent the agreed scores based on two independent reviewers' ratings. The six domains assessed were: Study Design (max 3), Sampling (max 3), Data Type (max 3), Validity Evidence (max 3), Data Analysis (max 4), and Study Outcomes (max 3). The total MERSQI score is the sum of the six-domain scores, with a maximum possible total of 18 points. Low methodological quality are scores between 1 – 6; moderate 7 –12 and high 13 – 18. Studies that are moderate and high quality are in bold. Full scoring per rater and the adapted MERSQI rubric are presented in Supplementary Tables S2 and S3.*

| **Section and Topic** | **Item #** | **Checklist item** | **Location where item is reported** |
| --- | --- | --- | --- |
| **TITLE** | | |  |
| Title | 1 | Identify the report as a systematic review. | Title |
| **ABSTRACT** | | |  |
| Abstract | 2 | See the PRISMA 2020 for Abstracts checklist. | Abstract |
| **INTRODUCTION** | | |  |
| Rationale | 3 | Describe the rationale for the review in the context of existing knowledge. | Introduction - rationale |
| Objectives | 4 | Provide an explicit statement of the objective(s) or question(s) the review addresses. | Introduction – research questions |
| **METHODS** | | |  |
| Eligibility criteria | 5 | Specify the inclusion and exclusion criteria for the review and how studies were grouped for the syntheses. | Methods – Eligibility criteria |
| Information sources | 6 | Specify all databases, registers, websites, organisations, reference lists and other sources searched or consulted to identify studies. Specify the date when each source was last searched or consulted. | Methods - Search strategy |
| Search strategy | 7 | Present the full search strategies for all databases, registers and websites, including any filters and limits used. | Methods - Search strategy and supplementary file |
| Selection process | 8 | Specify the methods used to decide whether a study met the inclusion criteria of the review, including how many reviewers screened each record and each report retrieved, whether they worked independently, and if applicable, details of automation tools used in the process. | Methods - Eligibility criteria and Search strategy |
| Data collection process | 9 | Specify the methods used to collect data from reports, including how many reviewers collected data from each report, whether they worked independently, any processes for obtaining or confirming data from study investigators, and if applicable, details of automation tools used in the process. | Methods - Eligibility criteria and Search strategy |
| Data items | 10a | List and define all outcomes for which data were sought. Specify whether all results that were compatible with each outcome domain in each study were sought (e.g. for all measures, time points, analyses), and if not, the methods used to decide which results to collect. | Methods – Data extraction |
|  | 10b | List and define all other variables for which data were sought (e.g. participant and intervention characteristics, funding sources). Describe any assumptions made about any missing or unclear information. | Methods – Data extraction |
| Study risk of bias assessment | 11 | Specify the methods used to assess risk of bias in the included studies, including details of the tool(s) used, how many reviewers assessed each study and whether they worked independently, and if applicable, details of automation tools used in the process. | Methods – Quality assessment (MERSQI) |
| Effect measures | 12 | Specify for each outcome the effect measure(s) (e.g. risk ratio, mean difference) used in the synthesis or presentation of results. | Methods – Data synthesis (narrative synthesis) |
| Synthesis methods | 13a | Describe the processes used to decide which studies were eligible for each synthesis (e.g. tabulating the study intervention characteristics and comparing against the planned groups for each synthesis (item #5)). | Methods – Data extraction using COM-B framework |
|  | 13b | Describe any methods required to prepare the data for presentation or synthesis, such as handling of missing summary statistics, or data conversions. | Methods – Data synthesis (COM-B framework) |
|  | 13c | Describe any methods used to tabulate or visually display results of individual studies and syntheses. | Methods – Table 1 COM-B definitions used for coding and supplementary file |
|  | 13d | Describe any methods used to synthesize results and provide a rationale for the choice(s). If meta-analysis was performed, describe the model(s), method(s) to identify the presence and extent of statistical heterogeneity, and software package(s) used. | Methods – Data synthesis (COM-B framework) |
|  | 13e | Describe any methods used to explore possible causes of heterogeneity among study results (e.g. subgroup analysis, meta-regression). | Not applicable - no meta-analysis due to heterogeneity acknowledged in Discussion. |
|  | 13f | Describe any sensitivity analyses conducted to assess robustness of the synthesized results. | Methods – Data synthesis (COM-B framework) |
| Reporting bias assessment | 14 | Describe any methods used to assess risk of bias due to missing results in a synthesis (arising from reporting biases). | Not reported |
| Certainty assessment | 15 | Describe any methods used to assess certainty (or confidence) in the body of evidence for an outcome. | Methods – Quality assessment (MERSQI) |
| **RESULTS** | | |  |
| Study selection | 16a | Describe the results of the search and selection process, from the number of records identified in the search to the number of studies included in the review, ideally using a flow diagram. | Results - Study selection and Figure 1 (PRISMA flow diagram) |
|  | 16b | Cite studies that might appear to meet the inclusion criteria, but which were excluded, and explain why they were excluded. | Results - Study selection (Excluded studies in flow diagram) |
| Study characteristics | 17 | Cite each included study and present its characteristics. | Results - Study characteristics and Table 2 |
| Risk of bias in studies | 18 | Present assessments of risk of bias for each included study. | Results - Quality of included studies and Supplementary file |
| Results of individual studies | 19 | For all outcomes, present, for each study: (a) summary statistics for each group (where appropriate) and (b) an effect estimate and its precision (e.g. confidence/credible interval), ideally using structured tables or plots. | Results - Individual studies (COM-B subsections, Tables/Figures) |
| Results of syntheses | 20a | For each synthesis, briefly summarise the characteristics and risk of bias among contributing studies. | Results - COM-B subsections |
|  | 20b | Present results of all statistical syntheses conducted. If meta-analysis was done, present for each the summary estimate and its precision (e.g. confidence/credible interval) and measures of statistical heterogeneity. If comparing groups, describe the direction of the effect. | Results - COM-B subsections  Figure 2 and supplementary tables |
|  | 20c | Present results of all investigations of possible causes of heterogeneity among study results. | Not applicable |
|  | 20d | Present results of all sensitivity analyses conducted to assess the robustness of the synthesized results. | Not conducted |
| Reporting biases | 21 | Present assessments of risk of bias due to missing results (arising from reporting biases) for each synthesis assessed. | Not applicable |
| Certainty of evidence | 22 | Present assessments of certainty (or confidence) in the body of evidence for each outcome assessed. | Results – quality of included studies using MERSQI |
| **DISCUSSION** | | |  |
| Discussion | 23a | Provide a general interpretation of the results in the context of other evidence. | Discussion |
|  | 23b | Discuss any limitations of the evidence included in the review. | Discussion – Limitations of the review |
|  | 23c | Discuss any limitations of the review processes used. | Discussion – Limitations of the review |
|  | 23d | Discuss implications of the results for practice, policy, and future research. | Discussion – Implication of the results |
| **OTHER INFORMATION** | | |  |
| Registration and protocol | 24a | Provide registration information for the review, including register name and registration number, or state that the review was not registered. | Methods: PROSPERO registration (CRD420250655653) |
|  | 24b | Indicate where the review protocol can be accessed, or state that a protocol was not prepared. | Methods: Protocol registration |
|  | 24c | Describe and explain any amendments to information provided at registration or in the protocol. | Discussion (amendments from protocol noted) |
| Support | 25 | Describe sources of financial or non-financial support for the review, and the role of the funders or sponsors in the review. | Funding section |
| Competing interests | 26 | Declare any competing interests of review authors. | Conflicts of interest section |
| Availability of data, code and other materials | 27 | Report which of the following are publicly available and where they can be found: template data collection forms; data extracted from included studies; data used for all analyses; analytic code; any other materials used in the review. | Data supplementary files |

*From:*  Page MJ, McKenzie JE, Bossuyt PM, Boutron I, Hoffmann TC, Mulrow CD, *et al.* The PRISMA 2020 statement: an updated guideline for reporting systematic reviews. BMJ 2021;372:n71. doi: 10.1136/bmj.n71. This work is licensed under CC BY 4.0. To view a copy of this license, visit <https://creativecommons.org/licenses/by/4.0/>
